# Chronic hypoxia protects the mouse heart from oxidative stress via HIF-1α-mitochondria crosstalk

**DOI:** 10.1007/s00018-026-06299-7

**Published:** 2026-06-18

**Authors:** Barbora Opletalova, Lukas Alan, Miroslav Ferko, Natalia Andelova, David Janko, Adam Eckhardt, Kristyna Holzerova, Romana Bohuslavova, Gabriela Pavlinkova, Frantisek Kolar, Marketa Hlavackova, Petra Alanova

**Affiliations:** 1 Institute of Physiology of the Czech Academy of Sciences, Prague, Czech Republic; 2 Department of Physiology, Faculty of Science, Charles University, Prague, Czech Republic; 3 Centre of Experimental Medicine, Institute for Heart Research, Slovak Academy of Sciences, Bratislava, Slovakia; 4 Institute of Biotechnology, Czech Academy of Sciences, Vestec, Czechia

**Keywords:** Cardioprotection, Chronic hypoxia, HIF-1α, Mitochondria, Permeability transition pore, Oxidative stress

## Abstract

Adaptation to chronic hypoxia (CH) enhances myocardial tolerance to ischemia/reperfusion injury and is closely associated with stabilization of hypoxia-inducible factor-1 alpha (HIF-1α), a central transcription factor in hypoxic response. Given the central role of mitochondria in cardiac pathophysiology, we investigated the contribution of HIF-1α to cellular mechanisms underlying CH-induced cardioprotection, with a focus on proteomic remodeling, antioxidant defense, and regulation of the mitochondrial permeability transition pore (mPTP). Adult male wild-type and heterozygous *Hif1a* knockout mice were exposed to intermittent CH (7000 m, 8 h/day, 4 weeks) or kept under normoxia. Isolated perfused hearts treated with cyclosporine A, an inhibitor of mPTP opening, were subjected to global ischemia/reperfusion insult for infarct size determination. Quantitative label-free proteomics was conducted to assess HIF-1α-dependent changes. We evaluated oxidative stress, measured levels of proteins associated with antioxidant defense and mPTP regulation. In parallel, a proof-of-concept study was performed in transfected AC16 cardiomyocytes exposed to H_2_O_2_-induced oxidative stress. CH induced HIF-1α-dependent cardioprotection by limiting infarct size through regulation of mPTP opening. Moreover, CH attenuated oxidative stress and promoted the protective translocation of hexokinase-2 to mitochondria in an HIF-1α-dependent manner. Consistently, HIF-1α overexpression enhanced cardiomyocyte survival under oxidative stress, whereas HIF-1α inhibition by acriflavine reduced cell viability. These findings identify HIF-1α as a key mediator of CH-induced adaptive response and cardioprotection during I/R injury in the mouse heart, acting through mPTP regulation.

**Graphical abstract:**

**Figure:**
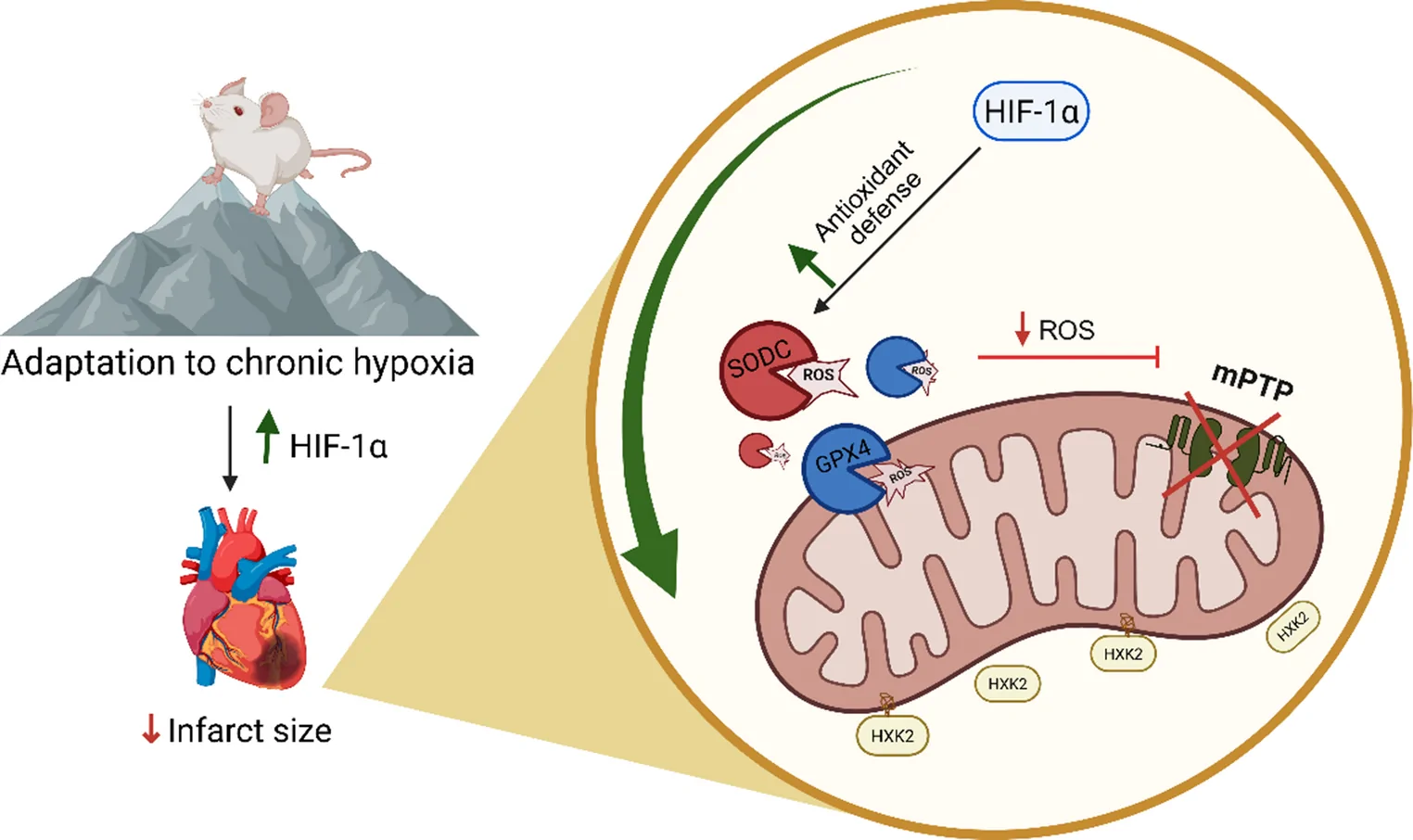
Cardioprotective effect of adaptation to chronic hypoxia (CH) is associated with the stabilization of hypoxia-inducible factor-1α (HIF-1α), a key regulator of hypoxic response. We explored the link between HIF-1α signaling and mitochondrial function in the CH-induced beneficial effect with respect to mitochondrial permeability transition pore (mPTP). HIF-1α acted through remodeling of proteome, increased expression of antioxidant enzymes (e.g., SODC, GPX4), cytoprotective translocation of hexokinase-2 (HXK2) to mitochondria, and modulation of mPTP opening. Noteworthy is that HIF-1α improved the cardiomyocyte survival as it enhanced antioxidant defense resulting in reactive oxygen species (ROS) reduction and mPTP opening regulation.

## Introduction

Ischemic heart disease is the leading cause of mortality and disability worldwide, manifesting clinically as acute myocardial infarction and ischemic cardiomyopathy. Myocardial ischemia/reperfusion (I/R) injury includes substantial changes in cardiac energy metabolism and performance, resulting in a series of events, such as ischemic and reperfusion arrhythmias, myocardial stunning, microvascular damage, cell death, and ultimately contractile dysfunction. While early restoration of coronary blood flow to the ischemic myocardium is crucial to prevent infarct enlargement, reperfusion itself may cause further cardiomyocyte death. Key factors include oxidative stress, calcium homeostasis disruption, and subsequent mitochondrial dysfunction [1].

Mitochondria occupy about one-third of the volume of adult cardiomyocytes, reflecting the myocardial high-energy demands. These organelles play a central role in both physiological heart function and disease, such as ischemic heart disease (for review, see [2]). During reperfusion, excessive production of reactive oxygen species (ROS) and intramitochondrial calcium overload trigger the permeability transition via prolonged opening of the large-conductance channel in the inner mitochondrial membrane (IMM), known as the mitochondrial permeability transition pore (mPTP) [3]. Sustained mPTP opening leads to disruption of oxidative phosphorylation, collapse of membrane potential, osmotic swelling of the mitochondrial matrix, mitochondrial rupture, and ultimately cell death [4]. The molecular structure of mPTP remains uncertain. Various proteins, such as voltage-dependent anion channel (VDAC) [5], mitochondrial phosphate carrier (MPCP) [6], Bcl-2-associated X protein (BAX) [7], and translocator protein (TSPO) [8], have been proposed to contribute to mPTP channel regulation. Recently, mitochondrial F_1_/F_O_-ATP synthase [9] and adenine nucleotide translocator (ANT) [10] have been implicated in pore formation, with matrix cyclophilin D (CYPD) facilitating the transition to the pore-forming conformation in a process modulated by calcium and ROS [11]. Notably, CYPD binding to the IMM is prevented by cyclosporine A (CsA) and by other molecules that interact with CYPD and are usually described as mPTP inhibitors [12].

Cardioprotection refers to interventions and mechanisms that contribute to reducing or even preventing myocardial damage. In the context of myocardial ischemia and infarction, adaptation to chronic hypoxia (CH) is the natural stimulus that confers long-lasting protection against all major end-points of I/R injury [13]. The key role in hypoxic signaling is mediated by the transcription factor hypoxia-inducible factor1 (HIF-1). HIF-1 works as a heterodimer consisting of an oxygen-regulated α subunit and a constitutive β subunit [14]. HIF-1 regulates over 1000 target genes involved in multiple events during hypoxia [15].

We recently showed that CsA administration did not further enhance the ischemic tolerance in rats adapted to CH, indicating that inhibition of mPTP opening contributes to the cardioprotective phenotype induced by CH [16]. In addition, we identified HIF-1α as an important mediator of the infarct-size-limiting effect induced by CH, acting by modulating mitochondrial dynamics and activating mitophagy [17]. However, the molecular mechanisms linking HIF-1α signaling to mPTP regulation remain unresolved. Given our previous findings and the established role of mPTP opening in controlling mitochondrial morphology and function [18], we hypothesized that HIF-1α modulates mPTP sensitivity during CH-induced cardioprotection. To address this hypothesis, we employed heterozygous *Hif1a* knockout mice and examined associated changes in proteomics, oxidative stress, and mitochondrial calcium handling.

## Materials and methods

### Animals and chronic hypoxia

The experiments were carried out in 12-week-old male wild-type (WT) mice and heterozygous *Hif1a* knockout mice (*Hif1a*^*+/−*^) carrying the *Hif1a*^*tm1jhu*^ null allele on the FVB background. *Hif1a*^*+/−*^ mice exhibit a reduced expression of the HIF-1α protein [19, 20]. Animals were maintained under controlled environmental conditions, with a stable room temperature of 22 ± 2 °C and *ad libitum* access to a standard laboratory diet (Altromin #1324, Germany) and water. Both WT and *Hif1a*^*+/−*^ mice were subjected to intermittent hypobaric hypoxia simulating an altitude of 7000 m above sea level (8 h/day, 5 days per week, 4 weeks) from the age of 8 weeks, as previously described [17]. Barometric pressure was gradually reduced in a stepwise manner, reaching the target altitude after 6 sessions. Mice were sacrificed on the day following the final hypoxic exposure. Control animals were maintained under normoxic conditions at room air.

### Isolated perfused hearts and infarct size determination

Animals were sacrificed by cervical dislocation. The hearts were promptly removed, cannulated via the aorta, and perfused *ex vivo* using the Langendorff apparatus under constant pressure (80 mmHg). A modified, non-recirculating Krebs-Henseleit buffer (118 mM NaCl, 25 mM NaHCO_3_, 4.7 mM KCl, 1.2 mM MgSO_4_, 1.2 mM KH_2_PO_4_, 2.5 mM CaCl_2_, 0.5 mM EDTA, 11 mM glucose; Penta Chemicals) was used, continuously gassed with a mixture of 95% O_2_ and 5% CO_2_ to maintain pH at 7.4 and kept at 37 °C. After 15 min of stabilization, the spontaneously beating hearts were subjected to 45 min of global normothermic no-flow ischemia, followed by 60 min of reperfusion. During ischemia, the hearts were submerged in Krebs-Henseleit buffer without glucose, gassed with N_2_. In half of the experiments, perfusion and anoxic buffers were supplemented with 0.2 µM CsA, an mPTP inhibitor targeting CYPD (Merck, C3662), or with a corresponding concentration of the solvent DMSO throughout the experiment.

At the end of reperfusion, the hearts were infused with a bolus of 2 mL of 1% 2,3,5-triphenyltetrazolium chloride (TTC; Merck, T8877) via the aorta, followed by incubation of the heart in TTC for 30 min at 37 °C. Subsequently, the atria were removed, and the hearts were fixed overnight in 10% neutral buffered formaldehyde at 4 °C. The following day, the right ventricle (RV) was separated, and the left ventricle (LV), including the septum, was sectioned into 5–6 slices transversely to its long axis. Two days after infarction, the slices were photographed and analyzed using computerized planimetric analysis (Ellipse software, ViDiTo, Slovakia). Infarct size was normalized to the total LV area.

### Tissue processing

In separate sets of experiments, normoxic and chronically hypoxic mice were sacrificed by cervical dislocation, their hearts were rapidly excised, washed in a cold PBS, and dissected into RV, LV, and the septum. All collected tissue segments were frozen in liquid nitrogen, weighed, and stored in liquid nitrogen until use.

### Proteomic analysis

Proteomic profiling of frozen LV tissue was performed using a nano-liquid chromatography system (Ultimate 3000 RSLC, Thermo Fisher Scientific, Germering, Germany) coupled to a mass spectrometer with electrospray ionization (ESI) and a 3D ion trap mass analyzer (Amazon SL, Bruker, Bremen, Germany). Tissue lysates containing 250 µg of protein were digested in solution with trypsin (Merck, USA) at a 1:25 enzyme-to-substrate ratio (0.2 µg/µL) overnight at 37 °C. Before liquid chromatography-mass spectrometry (LC-MS) analysis, samples were desalted using C18-U solid phase extraction columns (Strata, Phenomenex). The mobile phase consisted of component A (0.1% formic acid in 2% acetonitrile) and component B (0.1% formic acid in 95% acetonitrile). The loading phase consisted of 0.05% formic acid in 2% acetonitrile. The mobile phase flow rate was 0.3 µL/min with a gradient length of 290 min (4–7% B for 5 min, 7–30% B over 253 min, followed by 12 min at 95% B and 20 min at 4% B). Mass spectrometry was performed in positive ion mode with a scan range of 50–2200 m/z, capillary voltage of 1450 V, atomizer pressure of 0.4 Ψ, gas flow rate of 3.0 L/min, gas temperature of 150 °C, and a scan rate of 8100 m/z/s. The analyzed samples were separated on a 75 μm × 500 mm column (DNV PepMap TM Neo 1500 bar) with 2 μm particles at 100 Å and 40 °C. Additional LC-MS parameters, as well as detailed database search and protein identification procedures, were performed according to the protocol established by Andelova et al. [21].

### Oxidative stress assays

Markers of oxidative stress were measured in LV tissue. Levels of 3-nitrotyrosine (3-NT), a marker of nitrosative stress, were assessed using a commercially available ELISA kit (Abcam, ab116691) according to the manufacturer’s instructions. Briefly, tissue samples were homogenized in PBS and prepared using the provided extraction buffer. Samples and standards were incubated in a 96-well plate for 2 h. After two washing steps, the detection antibody was added and incubated for 1 h. Following two additional washes, an HRP-conjugated label was applied for 1 h. Finally, wells were washed three times, HRP Development Solution was added, and absorbance was measured at 600 nm over 15 min.

Lipid peroxidation was assessed by quantifying malondialdehyde (MDA) levels in LV tissue. Samples were homogenized using the Mixer Mill (Retsch, MM400) in a buffer containing 25 mM Tris-HCl and 0.1% Triton X-100, sonicated, and centrifuged at 1000 g for 10 min at 4 °C. To the supernatant, 20 µL of 6 M NaOH was added, followed by vortexing. Samples were incubated at 60 °C for 30 min, then cooled at -20 °C for 5 min. Proteins were precipitated by adding 50 µL of 35% (v/v) HClO_4_, followed by centrifugation at 10 000 g for 5 min at 4 °C. A 100 µL aliquot of the resulting supernatant was mixed with 10 µL of 5 mM 2,4-dinitrophenylhydrazine, incubated in the dark for 10 min, and analyzed by high-performance LC (Agilent 1100, US) using a Kinetex 100-2.6 LC Column (2.1 mm x 150 mm) with a flow rate of 0.28 mL/min and UV detection at 310 nm. Tissue contents of 3-NT and MDA were normalized to total protein content, determined by Bradford assay (Biorad, catalog no. 5000006).

### Mitochondrial isolation

For Western blotting, heart mitochondria were isolated in STE buffer (0.25 M sucrose, 10 mM Tris-HCl, 2 mM EDTA, pH 7.4) using a modified protocol of Pecinova et al. [22]. Briefly, the minced heart tissue (with removed atria) was homogenized with a teflon-glass homogenizer (7 strokes, 750 RPM), phosphatase inhibitors (Merck, P5726, 1:200), and protease inhibitors (Merck, P8340, 1:500) were added and centrifuged at high speed (8600 g, 10 min, 4 °C). The resulting pellet was resuspended in STE buffer, rehomogenized (7 strokes, 750 RPM, 4 °C), and inhibitors were added as in the previous step and centrifuged at low speed (600 g, 10 min, 4 °C). Supernatant was placed into a new Falcon tube and spun down (800 g, 10 min, 4 °C). Finally, the supernatant was transferred into a new Falcon tube and centrifuged at high speed (10 000 g, 10 min, 4 °C). The final pellet, containing mitochondria, and the supernatant, containing the cytosolic fraction, were collected and stored at -80 °C until used. Finally, the sample fractionation was verified by Western blot, visualizing representative cytosolic and mitochondrial proteins - vinculin and mitochondrial transcription factor A (TFAM), respectively (Fig. 6b).

For calcium retention capacity (CRC) measurement, heart mitochondria were isolated in TES buffer enriched with proteinase (bacterial proteinase type XXIV, Merck P8038) to receive functional mitochondria. Hearts were extracted and minced, then homogenized with teflon-glass homogenizer (4 strokes, 600 RPM) in TES buffer (100 mM sucrose, 50 mM KCl, 20 mM TES, 1 mM EDTA, 0.2% fatty acid free BSA, pH 7.2) containing proteinase (1 mg/g of the tissue) and centrifuged (8600 g, 10 min, 4 °C). Pellet was resuspended in TES buffer (without proteinase) containing protease inhibitors (Merck, P8340, 1:500), rehomogenized by hand with a teflon-glass homogenizer (4 strokes), and centrifuged (800 g, 10 min, 4 °C). Supernatant was placed into a new Falcon tube and spun down (8600 g, 10 min, 4 °C). The final mitochondrial pellet was resuspended in TES buffer, and total protein content was measured by Bradford assay.

### Calcium retention capacity

Freshly isolated mitochondria were transferred to a 48-well plate with 0.5 mL of CRC measurement medium [0.1 M sucrose, 80 mM KCl, 4 mM MgCl_2_, 10 mM MOPS-Tris buffer (pH 7.2), 5 mM succinate, 1 mM potassium phosphate-Tris buffer (pH 7.4), 10 µM EGTA, 2 µM rotenone] per well. The final pH of the medium was adjusted to 7.2. Subsequently, 0.5 µM Calcium Green™-5 N fluorescent probe (Thermo Fisher, C3737) and either 1 µM CsA or ethanol (vehicle control) were added to each well. The samples were analyzed using the Infinite M200 plate reader (Tecan, Germany) with an excitation wavelength of 505 nm (bandpass 9 nm) and an emission wavelength of 535 nm (bandpass 20 nm). Measurements were recorded for 100 cycles, with each cycle lasting 30 s. Every 5th cycle, 10 µL of 0.5 mM CaCl_2_ was added to each well.

### Cell culture and treatment

The proliferating AC16 cell line (Merck, SCC109), generated from adult human ventricular cardiomyocytes, was maintained in growth medium DMEM/Nutrient Mixture F-12 (Thermo Fisher Scientific, 11320033) supplemented with 10% (vol/vol) FBS (Thermo Fisher Scientific, 10270-106) and antibiotics (100 U/mL penicillin + 100 µg/mL streptomycin; Thermo Fisher Scientific, 15140-122) at 37 °C in a humidified atmosphere of 5% CO_2_ [23]. Cells were subcultured until 70% confluence, then divided. Cells were transfected either with proline hydroxylases resistant HA-HIF1alpha P402A/P564A-pcDNA3 plasmid (Addgene, 18955) or empty vector (EV) pcDNA3.1 by GenJet In Vitro DNA Transfection Reagent (MerckGen Laboratories, SL100489), resulting in HIF-1α overexpression (Fig. 5c). For Western blotting, cell lysates were prepared 72 h after transfection. For cell death measurement, cells were placed in a 24-well plate 48 h after transfection. Attached cells were untreated or treated with 1 µM HIF-1 inhibitor acriflavine (ACF; Merck, A8251) for 15 h. Cells with 60% confluence were treated with 200 µM H_2_O_2_ for 4 h, and cell death was measured by lactate dehydrogenase (LDH) release into the supernatant using the Cytotoxicity Detection Kit (Roche, 11644793001) according to the manufacturer´s instructions.

### Western blotting

The levels of mitochondrial proteins were analyzed in mitochondria isolated from hearts with the atria removed. In the case of hexokinase-2 (HXK2), detection was also performed in the cytosolic fraction. Other cytosolic proteins were assessed in the LV tissue homogenates.

For homogenate preparation, LV tissue was homogenized using a TissueLyser in eight volumes of ice-cold homogenization buffer [12.5 mM Tris (pH 7.4), 250 mM sucrose, 2.5 mM EGTA, 1 mM EDTA, 6 mM β-mercaptoethanol] supplemented with protease (Merck, 04693159001) and phosphatase (Merck, 04906837001) inhibitors. The homogenates were centrifuged at 10 000 g for 10 min at 4 °C [17]. Isolated mitochondria and cytosolic fraction samples were lysed using RIPA buffer (Merck, 20-188) containing protease inhibitors (Merck, P8340, 1:500). Samples were incubated on ice for 30 min with frequent vortexing and sonication (at least three cycles). Lysates were subsequently centrifuged at 12 000 g for 15 min at 4 °C. Supernatants were collected and stored at -80 °C until use. Total protein concentrations were determined using the Bradford assay.

Protein samples were separated by SDS-PAGE using 4- 12% NuPAGE™ Bis-Tris gels (Invitrogen, NP0322BOX), and subsequently transferred onto polyvinylidene difluoride membranes (Bio-Rad, 1620177). Membranes were blocked for 1 h at room temperature in 5% non-fat dry milk diluted in Tris-buffered saline with 0.1% Tween-20. After washing, membranes were incubated overnight at 4 °C with the following primary antibodies: BAX (Abcam, ab32503, 1:500), catalase (CAT; Abcam, ab16731, 1:2000), CYPD (Abcam, ab110324, 1:1000), glutathione peroxidase 4 (GPX4; Abcam, ab125066, 1:5000), HIF-1α (Novus Biologicals, NB100-479, 1:1000), HXK2 (Abcam, ab209847, 1:1000), LDH (Abcam, ab52488, 1:10 000), mitochondrial calcium uniporter (MCU; Antibodies, A89422, 1:500), mitochondrial calcium uptake 1 (MICU1; Merck, HPA037480, 1:500), cytosolic superoxide dismutase (SODC; Cayman, 10011387, 1:5000), mitochondrial superoxide dismutase (SODM; Cayman, 10011390, 1:2000), TFAM (Abcam, ab119684, 1:1000), VDAC (a gift from Prof. Vito De Pinto, University of Catania, Italy, 1:1000). Following overnight incubation, membranes were washed and incubated for 1 h at room temperature with fluorescent secondary antibodies Alexa Fluor 680 (Invitrogen, A10038 and A10043, 1:10 000) and IRDye 800CW (LI-COR, 926-32212, 1:15 000). Bands were visualized using the Odyssey CLx imaging system (LI-COR Biosciences, Lincoln, NE, USA) and quantified with ImageJ software (NIH, Java Technology, Cupertino, CA, USA). Samples from each experimental group were run on the same gel and quantified on the same membrane to ensure consistency. As loading controls, vinculin (Merck, V9264, 1:5000) was used for tissue homogenate samples, and Ponceau S (Merck, 6226-79-5) was used for mitochondrial samples.

### Statistical analysis

Statistical analyses were performed using GraphPad Prism 8 software (GraphPad Inc., CA, USA). Two-way ANOVA was used to assess the effects of genotype and experimental conditions. When treatment was included, a three-way ANOVA was used. Significant interactions were followed by Bonferroni´s *post hoc* test for multiple comparisons. Comparisons of two groups were performed using an unpaired Student´s *t* test. Data are presented as means ± SEM or SD (*n* < 7). *p*-value < 0.05 was considered statistically significant.

For the proteomic dataset, protein abundance was estimated using emPAI values, which were log_2_-transformed before statistical analysis. Differential abundance between groups was assessed using Student’s *t* test or a non-parametric alternative, depending on data distribution. For exploratory analysis, *p*-values were not adjusted for multiple comparisons to enable identification of potentially relevant candidate proteins. Results were interpreted in the context of overall expression trends and biological relevance. Gene Ontology (GO) enrichment analysis was performed using the STRING database. Enrichment significance was evaluated using false discovery rate (FDR), with *p*-values corrected for multiple testing using the Benjamini-Hochberg procedure. Heatmaps were generated using MORPHEUS to visualize protein expression patterns across experimental groups. Volcano plots were used to assess differential protein abundance between conditions. Protein-protein interaction (PPI) networks were constructed using the STRING database and visualized in Cytoscape.

## Results

### Chronic hypoxia inhibited mPTP opening via HIF-1α

**Fig. 1:**
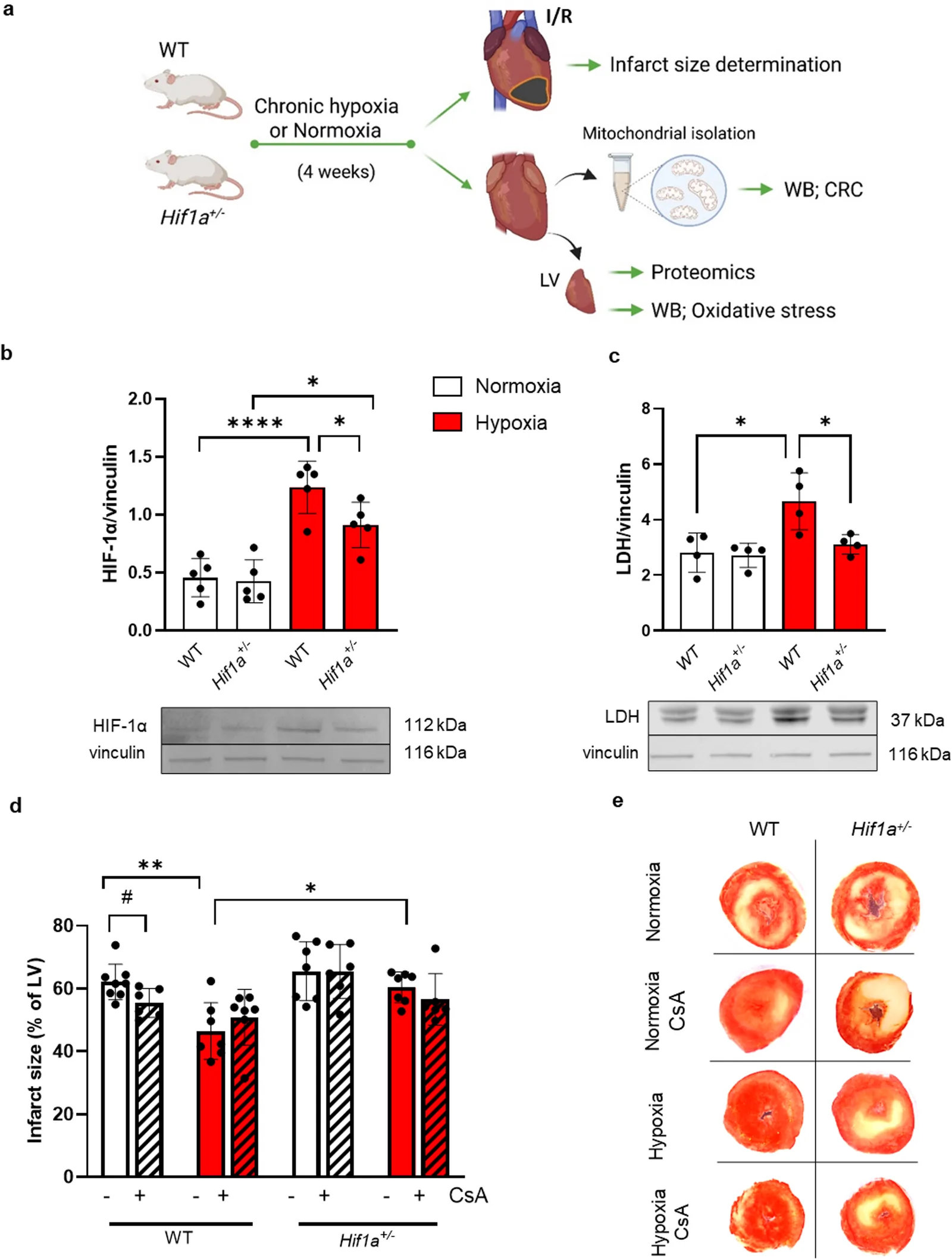
The effect of chronic hypoxia and CsA treatment on myocardial ischemic tolerance. **a** Experimental design: WT and *Hif1a*^*+/−*^ mice were adapted to CH or kept in normoxia for 4 weeks. At the end of the adaptation period, all 4 experimental groups were (i) subjected to ischemia/reperfusion (I/R) insult for infarct size determination or (ii) myocardial samples were collected for different analyses. Created with BioRender.com. Myocardial protein levels of (**b**) hypoxia-inducible factor-1 alpha (HIF-1α) and (**c**) lactate dehydrogenase (LDH) in tissue homogenates normalized to vinculin in the left ventricle (LV) of normoxic and chronically hypoxic WT and *Hif1a*^*+/−*^ mice. Values are means ± SD, *n* = 4 mice per group, *p < 0.05, ****p < 0.0001, two-way ANOVA with Bonferroni’s multiple comparisons test. (**d)** Infarct size normalized to the size of the LV of normoxic and chronically hypoxic WT and *Hif1a*^*+/−*^ hearts, untreated or treated with CsA. Values are means ± SD, *n* = 6–8 mice per group, *p < 0.05, **p < 0.01, three-way ANOVA with Bonferroni’s multiple comparisons test; #*p* = 0.037, Student´s unpaired *t* test. (**e)** Representative images of histochemically stained cross sections of the LV from normoxic and chronically hypoxic WT and *Hif1a*^*+/−*^ mice, untreated or treated with CsA. Red color represents survived tissue, and pale represents infarcted tissue

To assess whether HIF-1α is involved in the regulation of mPTP opening and how important this is for the cardioprotective effect of CH, we subjected the untreated or CsA-treated hearts of normoxic and chronically hypoxic WT and *Hif1a*^*+/−*^ mice to I/R insult and compared infarct size among the experimental groups (Fig. 1a). We analyzed the content of HIF-1α and its target LDH in the LV of normoxic and chronically hypoxic WT and *Hif1a*^*+/−*^ mice. WT mice responded to CH with the increased levels of HIF-1α (Fig. 1b) and LDH (Fig. 1c). Our results confirmed the infarct size-limiting effect of CH, as chronically hypoxic WT mice demonstrated reduced infarct size compared to their normoxic controls. This reduction was also significant when compared to chronically hypoxic *Hif1a*^*+/−*^ mice, suggesting that this beneficial effect was HIF-1α-dependent. CsA administration reduced infarct size in normoxic WT hearts compared to untreated controls; although this effect did not reach significance by three-way ANOVA, it was significant when assessed by the unpaired Student´s *t* test (*p*-value = 0.037). In contrast, CsA did not further enhance the protective effect in chronically hypoxic hearts (Fig. 1d and e). Altogether, both CH and CsA administration reduced infarct size in WT mice only, while the effect was abolished in mice with partial HIF-1α deficiency. These results highlight the importance of HIF-1α signaling in regulating mPTP opening during cardioprotection.

### Chronic hypoxia induced proteomic changes in the heart

**Fig. 2:**
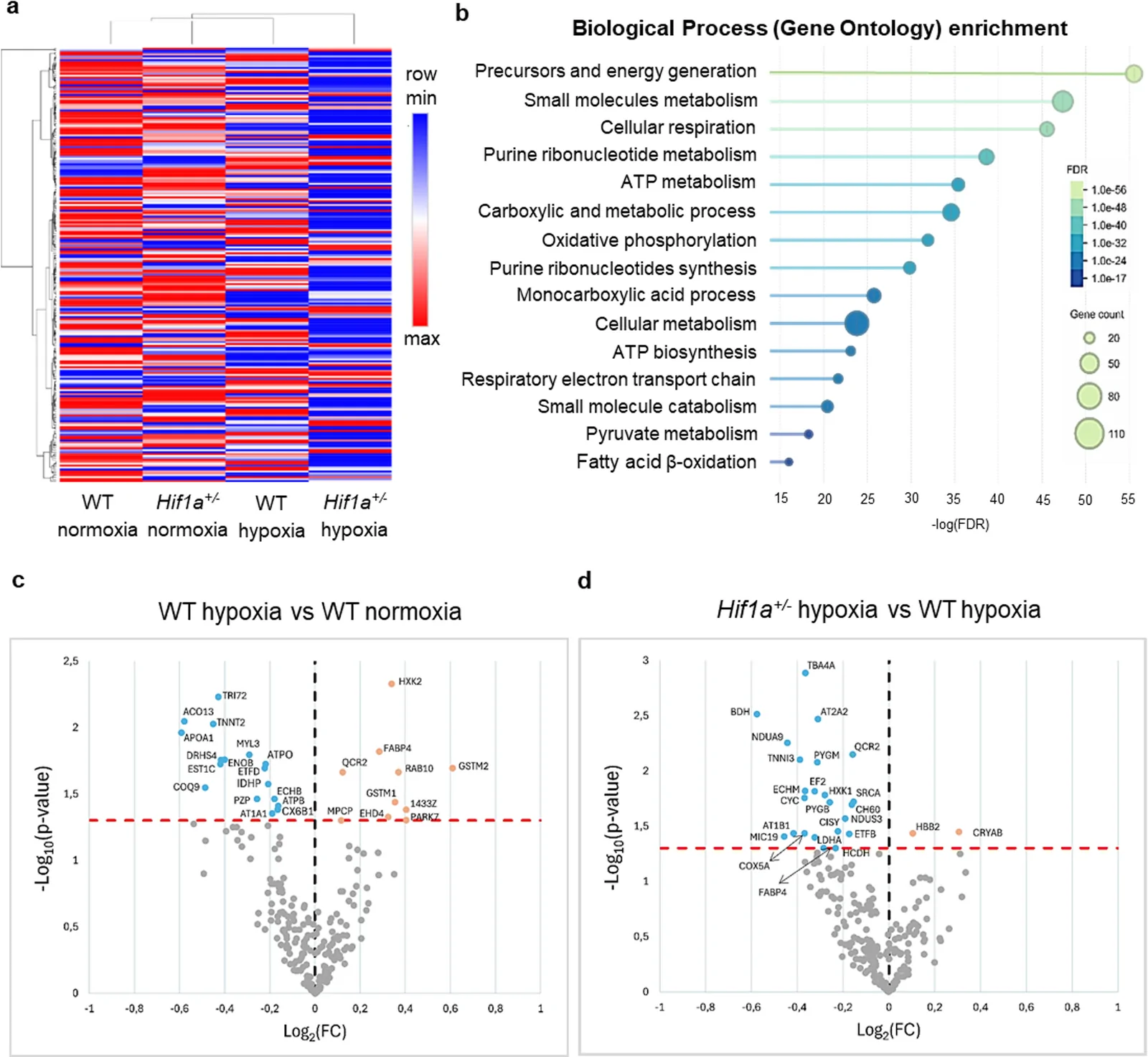
Characterization of proteomic changes induced by chronic hypoxia. (**a)** Heatmap showing protein expression profiles across experimental groups. Each row represents a protein and each column a sample group; color scale indicates relative expression (blue, low; red, high). (**b)** Gene Ontology (GO) enrichment analysis of downregulated proteins (*Hif1a*^*+/−*^ hypoxia vs. WT hypoxia) performed using STRING. Biological processes are ranked according to enrichment significance (FDR-adjusted). (**c**, **d)** Volcano plots showing differential protein abundance between (**c**) WT hypoxia vs. WT normoxia and (**d**) *Hif1a*^*+/−*^ hypoxia vs. WT hypoxia. Each point represents a protein, plotted as log_2_ fold change (FC) versus - log_10_ *p*-value. *p*-values are unadjusted and are presented as part of the discovery phase analysis

To gain insights on how CH and partial *Hif1a* deficiency affect the heart at the proteomic level, we performed MS-based quantitative proteomic analysis of the LV myocardium of normoxic and chronically hypoxic WT and *Hif1a*^*+/−*^ mice (Fig. 1a). We identified 266 differentially expressed proteins (Fig. 2), representing only those falling within a shared intersection of intergroup comparison. The clustering analysis revealed proteomic changes induced by adaptation to CH and more evidently by partial *Hif1a* deletion (Fig. 2a). Focusing on the predominant HIF-1α-induced differences between chronically hypoxic *Hif1a*^*+/−*^ and WT hearts, GO enrichment analysis displayed downregulated proteins in specific GO term categories associated with metabolic processes, aerobic respiration, and oxidative phosphorylation (Fig. 2b). Regarding proteins related to CH-induced changes (Fig. 2c), we identified 10 significantly upregulated proteins linked with cell protection against oxidative stress (GSTM1, GSTM2, HXK2, PARK7), HIF-1α signaling (FABP4, HXK2), cellular respiration (QCR2), membrane reorganization (EHD4, RAB10), and mitochondrial phosphate transport (MPCP) in chronically hypoxic WT group. In this group, we also observed downregulation of proteins involved in muscle contraction (MYL3, TRI72, TNNT2), fatty acid oxidation (ACO13, ETFD, ECHB), lipid metabolism (APOA1), and oxidative phosphorylation (ATPO, ATPB, COQ9, CX6B1). The only downregulated glycolytic enzyme was ENOB. We also compared the proteome of chronically hypoxic WT and *Hif1a*^*+/−*^ hearts to reveal specific differences in cellular responses (Fig. 2d). Chronically hypoxic *Hif1a*^*+/−*^ hearts exhibited downregulation of proteins linked with ketone metabolism (BDH); HIF-1α signaling (LDHA, FABP4); mitochondrial electron transport chain (CYC, COX5A, NDUA9 and NDUS3); the MICOS complex (MIC19); TCA cycle (CISY) and metabolism (HXK1, HCDH). Upregulated were only two proteins, including CRYAB, a molecular chaperone that primarily binds misfolded proteins to prevent protein aggregation under a wide range of stress conditions, and hemoglobin subunit HBB2.

The observed protein changes primarily served to highlight biological pathways and functional processes potentially affected by the experimental model. These findings subsequently guided downstream analyses, where selected targets and pathways were further validated using independent experimental approaches. Specifically, key proteins involved in antioxidant defense and mitochondrial regulation were assessed by Western blot analysis in LV tissue and isolated mitochondrial fractions. In addition, functional readouts including oxidative stress markers, mitochondrial calcium handling, and infarct size were evaluated. Furthermore, the role of HIF-1α was confirmed in a complementary cell model using gain- and loss-of-function approaches. The consistency between proteomic trends and these orthogonal measurements supports the reliability of the observed pathway-level changes.

### Chronic hypoxia reduced oxidative stress

**Fig. 3:**
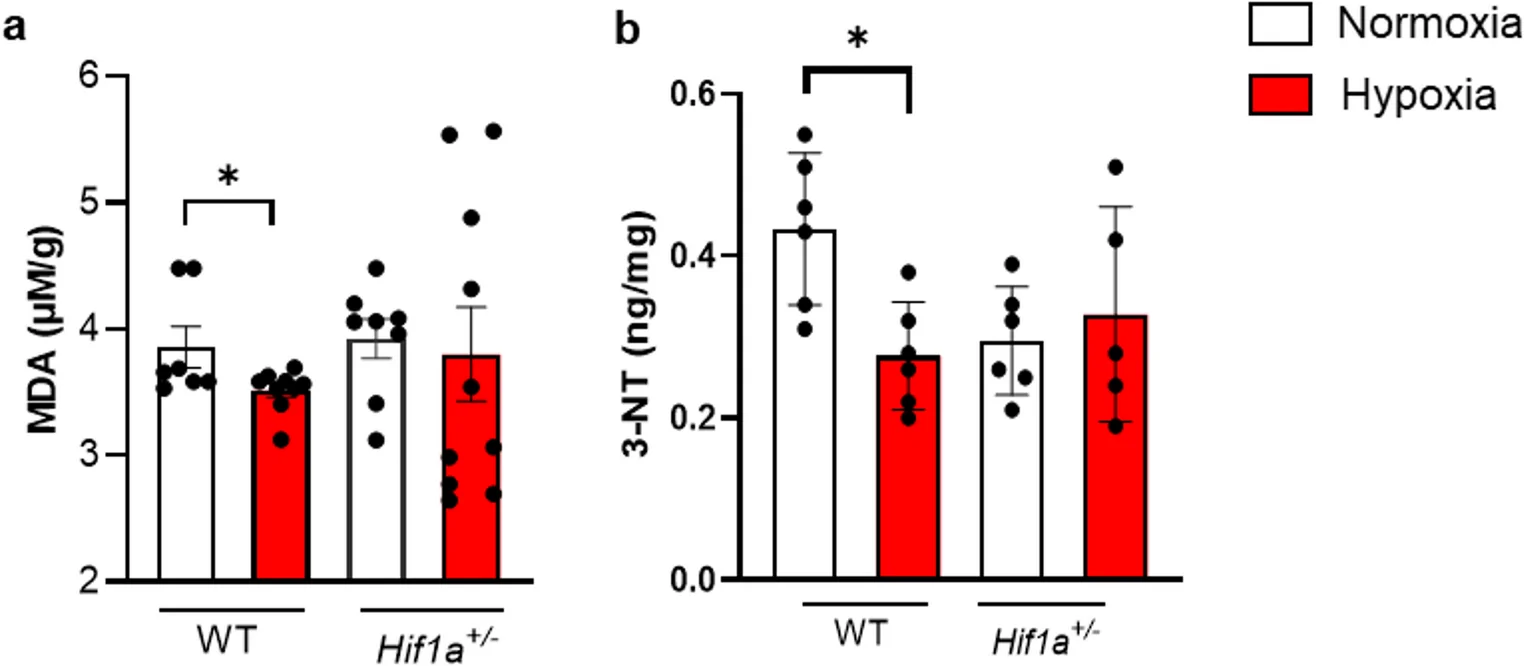
Adaptation to chronic hypoxia reduced levels of oxidative stress markers. Myocardial concentrations of (**a**) malondialdehyde (MDA) and (**b**) 3-nitrotyrosine (3-NT) in the left ventricle of normoxic and chronically hypoxic WT and *Hif1a*^*+/−*^ mice. Values are means ± SEM (**a**) or SD (**b**), *n* = 5–10 mice per group, *p < 0.05, unpaired Student´s *t* test (**a**), two-way ANOVA with Bonferroni’s multiple comparisons test (**b**)

**Fig. 4:**
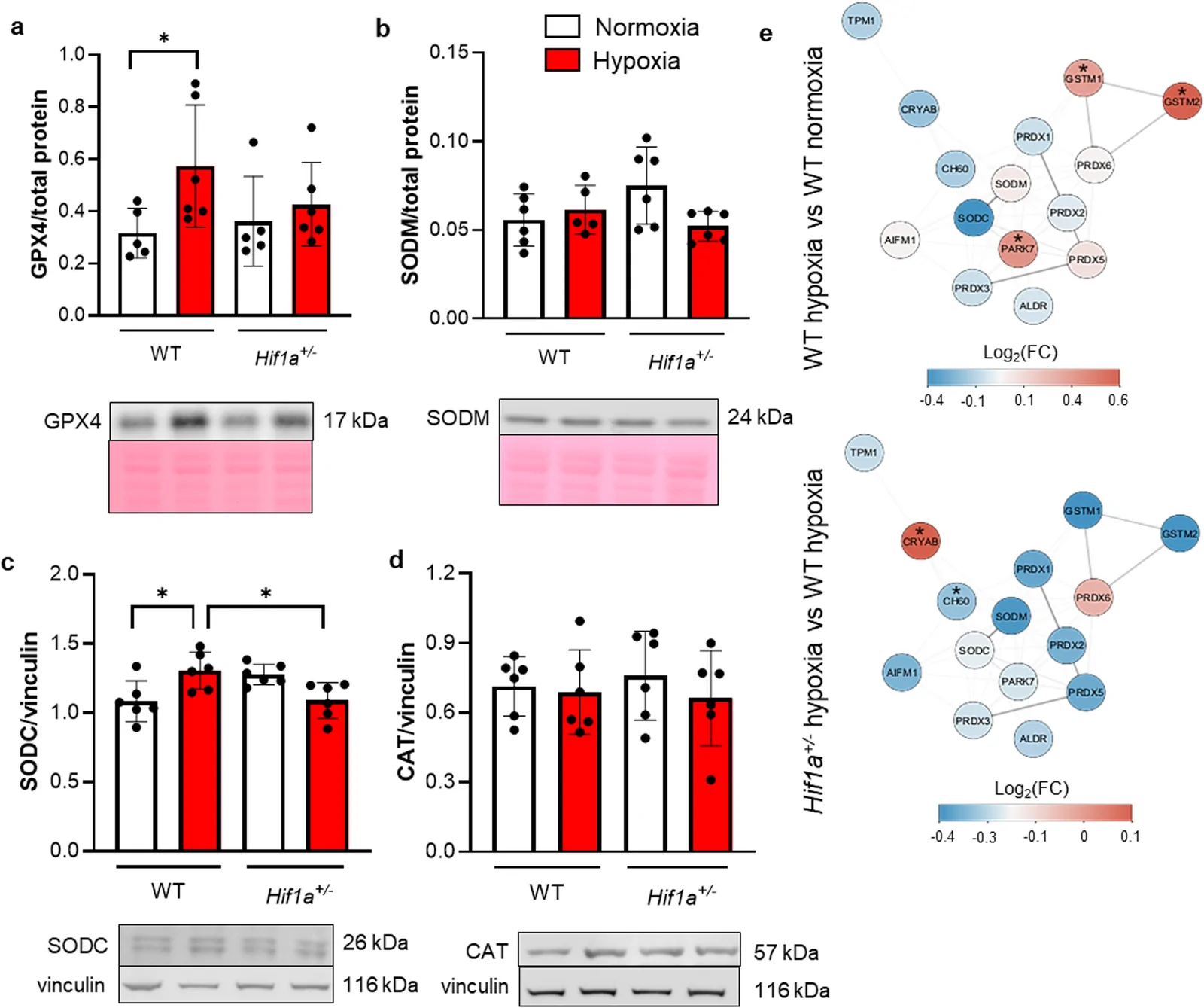
Adaptation to chronic hypoxia enhanced antioxidant defense. Myocardial protein levels of (**a**) glutathione peroxidase 4 (GPX4) and (**b**) mitochondrial superoxide dismutase (SODM) in isolated mitochondria normalized to total protein, and (**c**) cytosolic superoxide dismutase (SODC) and (**d**) catalase (CAT) in tissue homogenates normalized to vinculin in the left ventricle of normoxic and chronically hypoxic WT and *Hif1a*^*+/−*^ mice. Values are means ± SD, *n* = 5–6 mice per group, *p < 0.05, unpaired Student‘s *t* test (**a**), two-way ANOVA with Bonferroni’s multiple comparisons test (**c**). (**e**) Protein-protein interaction (PPI) networks of proteins associated with the response to reactive oxygen species under hypoxic conditions. Networks were generated using STRING and visualized in Cytoscape. Nodes represent proteins and are colored according to log_2_ fold change (FC; red, upregulated; blue, downregulated). Edges indicate predicted protein-protein associations (minimum interaction confidence score 0.4). Proteins with *p* < 0.05 are marked with an asterisk (*). Upper panel: WT hypoxia vs. WT normoxia; lower panel: *Hif1a*^*+/−*^ hypoxia vs. WT hypoxia

Since oxidative stress is a well-established trigger of mPTP opening, we examined the key markers - MDA, a marker of lipid peroxidation, and 3-NT, a marker of protein oxidation, in the LV myocardium of normoxic and chronically hypoxic WT and *Hif1a*^*+/−*^ mice (Fig. 1a). CH significantly decreased both markers in chronically hypoxic WT mice compared to their corresponding normoxic control groups (Fig. 3a and b). To determine whether CH enhances the antioxidant response, we analyzed the protein levels of major mitochondrial and cytosolic antioxidant enzymes (Fig. 4). The levels of both GPX4 (Fig. 4a) and SODC (Fig. 4c) were elevated in WT groups after adaptation to CH. In the case of SODC, this increase was HIF-1α-dependent. In contrast, SODM and CAT levels did not show any significant changes (Fig. 4b and d).

These findings are consistent with our proteomic data as a STRING network analysis of differentially expressed proteins identified 15 proteins involved in the response to oxidative stress (with node color intensity proportional to fold change, Fig. 4e). In WT hearts, adaptation to CH significantly upregulated glutathione S-transferase Mu 1 and 2 (GSTM1, GSTM2) and Parkinson disease protein 7 homolog (PARK7). There was a trend toward upregulation of peroxiredoxins 5 and 6 (PRDX5, PRDX6). In chronically hypoxic *Hif1a*^*+/−*^ hearts, most of these proteins were downregulated, except for CRYAB, which was significantly upregulated, and PRDX6, which showed an increasing trend compared to chronically hypoxic WT hearts.

**Fig. 5:**
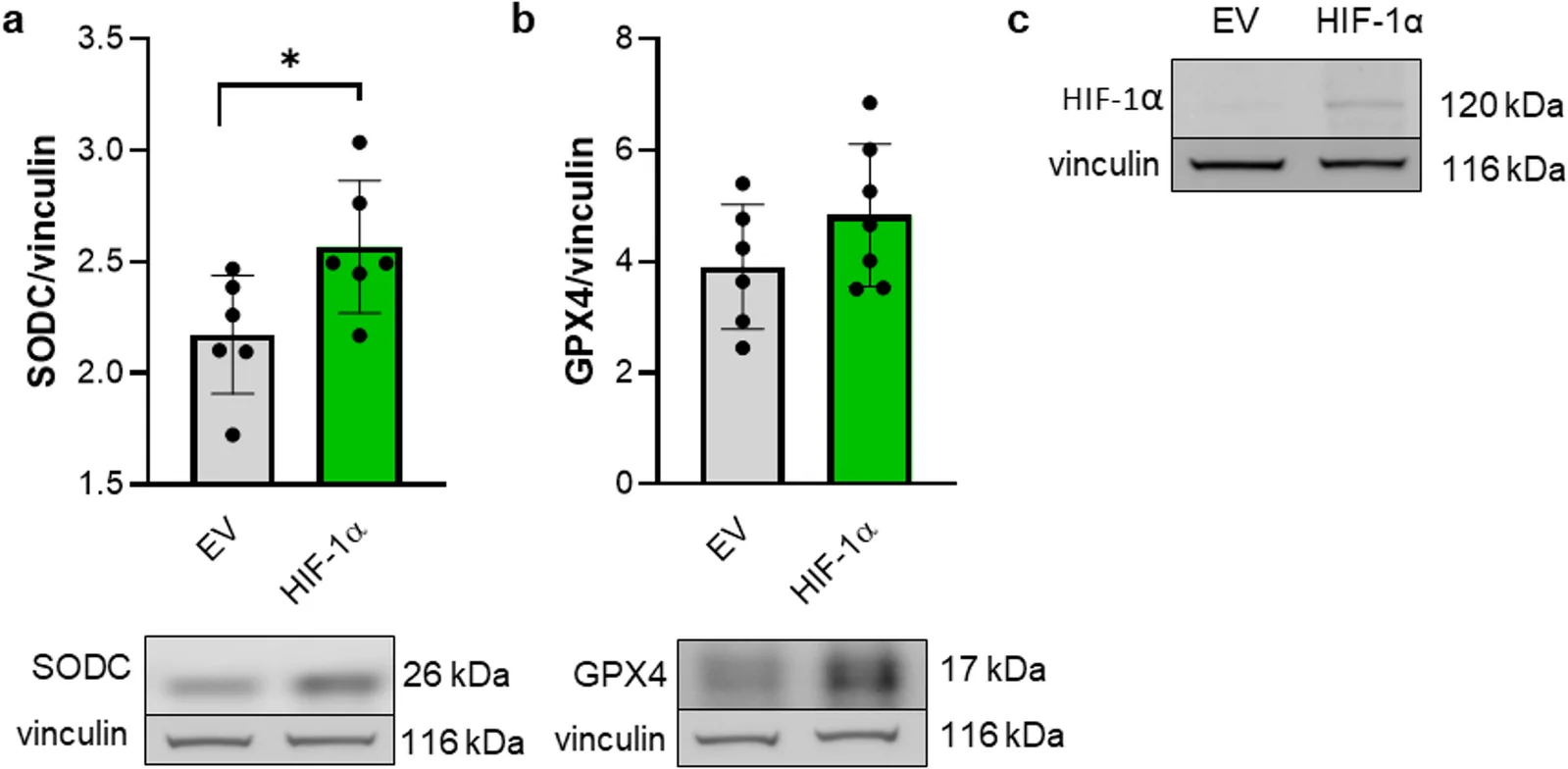
HIF-1α overexpression elevated the protein levels of antioxidant enzymes in the AC16 cell line. Protein levels of (**a**) cytosolic superoxide dismutase (SODC) and (**b**) glutathione peroxidase 4 (GPX4) normalized to vinculin in the AC16 cells transfected with empty vector (EV) or HIF-1α overexpressing plasmid (HIF-1α). Values are means ± SD, *n* = 6, *p < 0.05, unpaired Student´s *t* test. (**c**) Representative western blots in the AC16 cell line with EV or HIF-1α overexpression

We also verified our findings in a cell model using the immortalized AC16 human cardiomyocyte cell line, transfected either with EV or an HIF-1α overexpression vector. HIF-1α overexpression resulted in enhanced content of SODC (Fig. 5a) and an increasing trend in GPX4 (Fig. 5b). The efficiency of transfection was verified by the increased level of HIF-1α protein in HIF-1α overexpressing cells compared to EV cells (Fig. 5c).

### Chronic hypoxia changed the level of mPTP-related proteins

**Fig. 6:**
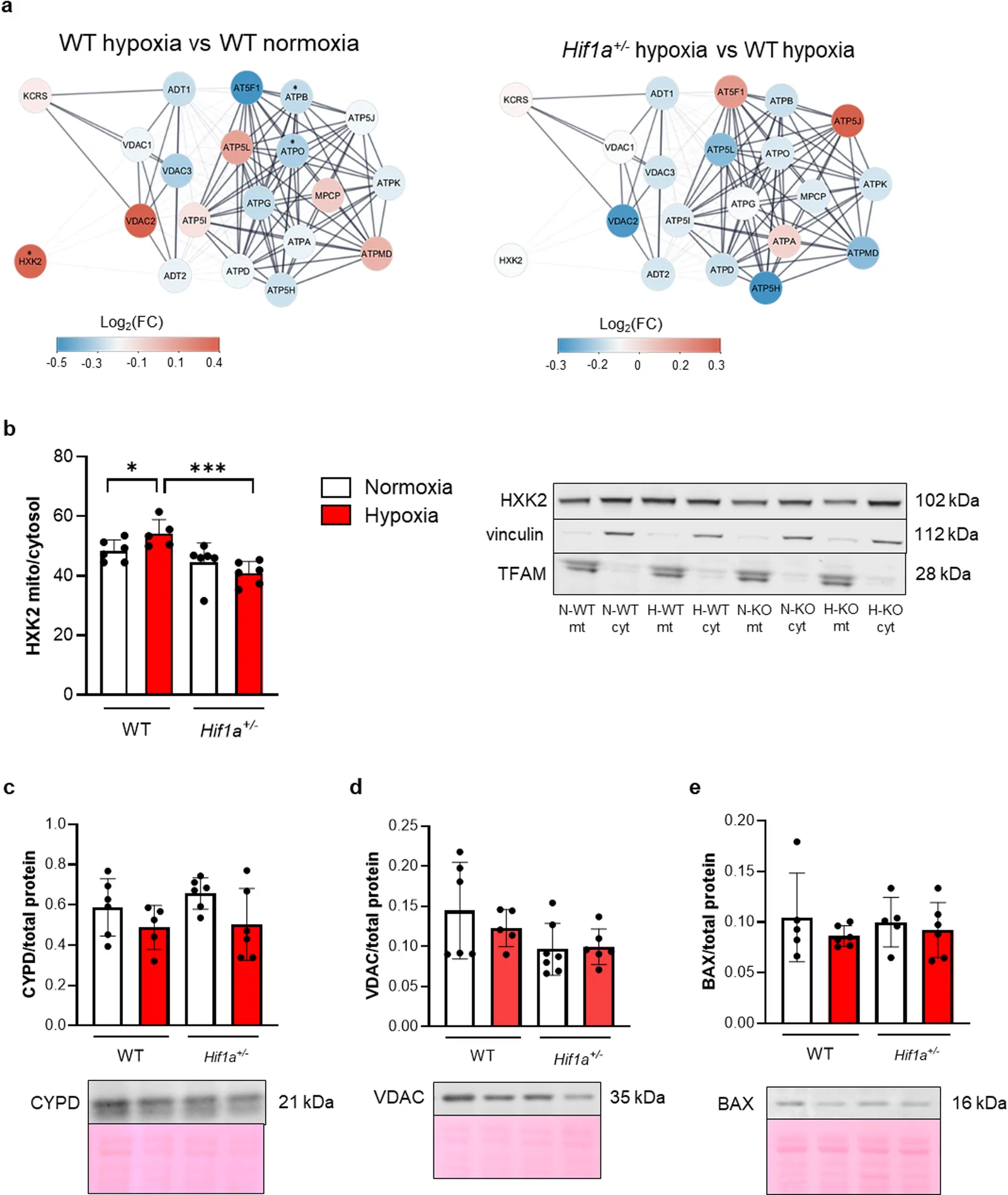
Adaptation to chronic hypoxia partially influenced mPTP-associated proteins. (**a**) Protein-protein interaction (PPI) networks of proteins associated with mPTP regulation. Networks were generated using STRING and visualized in Cytoscape. Nodes represent proteins and are colored according to log_2_ fold change (FC; red, upregulated; blue, downregulated). Edges indicate predicted protein-protein associations (minimum interaction confidence score 0.4). Proteins with *p* < 0.05 are marked with an asterisk (*). Left panel: WT hypoxia vs. WT normoxia; right panel: *Hif1a*^*+/−*^ hypoxia vs. WT hypoxia. (**b**) Ratio of hexokinase-2 (HXK2) protein levels in mitochondrial and cytosolic fractions prepared from hearts of normoxic and chronically hypoxic WT and *Hif1a*^*+/−*^ mice. Myocardial protein levels of (**c**) cyclophilin D (CYPD), (**d**) voltage-dependent anion channel (VDAC), and (**e**) Bcl-2-associated X protein (BAX) in isolated mitochondria normalized to total protein. Values are means ± SD, *n* = 5–7 mice per group, *p < 0.05, two-way ANOVA with Bonferroni’s multiple comparisons test (**b**)

To investigate possible cardioprotective mechanisms at the mPTP level, we constructed PPI networks and examined the interactions between putative mPTP-forming and mPTP-regulating proteins (Fig. 6). Our proteomic analysis identified 20 mPTP-related proteins that were altered across the experimental groups. Based on available data, these proteins implicated in the structure and regulation of mPTP were grouped into functional classes [24] to visualize their PPI and content. In WT hearts, adaptation to CH significantly increased HXK2 levels and reduced ATPB and ATPO levels. There was also a trend toward increased levels of VDAC2, KCRS, MPCP, and subunits of F_1_/F_O_-ATP synthase (ATP5I, ATP5L, ATPMD). In contrast, the level of proteins including AT5F1, VDAC1 and 3, ADT1 and 2, and subunits of F_1_/F_O_-ATP synthase (ATPG, ATP5H) tended to decrease (Fig. 6a). In chronically hypoxic *Hif1a*^*+/−*^ hearts (compared to chronically hypoxic WT hearts), protein levels of ATP5J, AT5F1, ATPA were upregulated, while protein levels of VDAC2, ATP5H, ATP5I, ATP5L, ATPMD were downregulated; however, these changes were not statistically significant.

The content of mPTP-regulating proteins was also measured by Western blotting. We detected HXK2 protein level, which has also been implicated in regulating mPTP function [25]. Under physiological conditions, HXK2 is primarily cytosolic; however, it translocates to the outer mitochondrial membrane under stress; such as severe hypoxia, to mediate cytoprotection [26, 27]. We measured HXK2 distribution between mitochondrial and cytosolic fractions and found that the mitochondrial-to-cytosolic ratio was significantly increased in chronically hypoxic WT hearts compared to normoxic controls as well as chronically hypoxic *Hif1a*^*+/−*^ hearts (Fig. 6b). However, another detected proteins, such as CYPD, VDAC, and BAX were not significantly changed across all experimental groups (Fig. 6c, d and e).

Therefore, we can conclude that CH influences putative mPTP-forming proteins, such as ATPase subunits, and affects mPTP-regulating proteins, especially HXK2, whose mitochondrial translocation was significantly increased in the presence of HIF-1α.

### HIF-1α affected the mitochondrial calcium uniporter

**Fig. 7:**
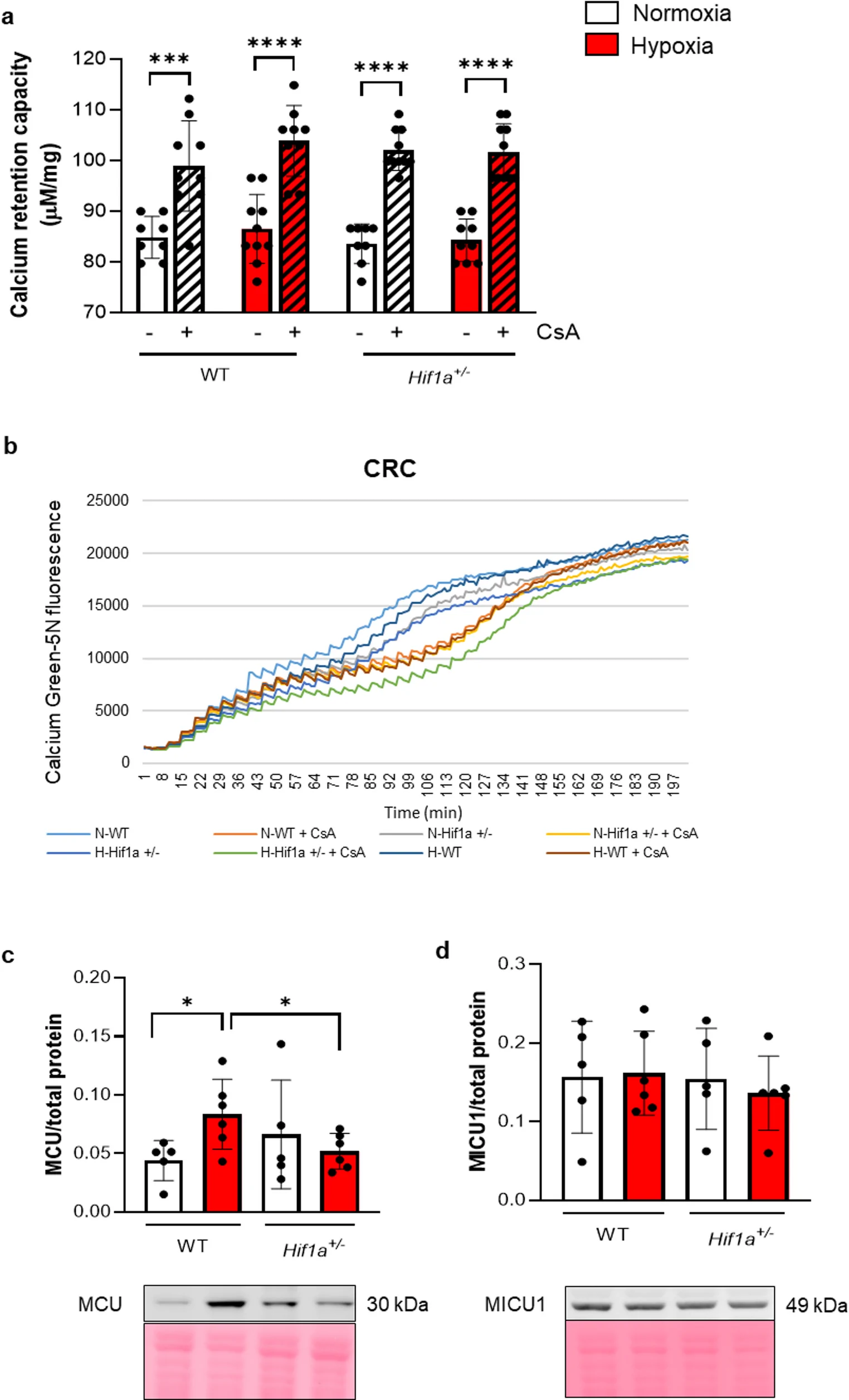
HIF-1α affected the mitochondrial calcium uniporter. (**a**) Mitochondrial calcium retention capacity (CRC) performed in mitochondria untreated or treated with CsA, which were isolated from normoxic and chronically hypoxic WT and *Hif1a*^*+/−*^ hearts. Values are means ± SEM, ***p < 0.001, ****p < 0.0001, *n* = 9–10 per group, three-way ANOVA with Bonferroni’s multiple comparisons test. (**b**) Representative curves of CRC. (**c**) Protein levels of mitochondrial calcium uniporter (MCU) and (**d**) mitochondrial calcium uptake 1 (MICU1, MCU regulator) in mitochondria isolated from normoxic and chronically hypoxic WT and *Hif1a*^*+/−*^ hearts. Values are means ± SD, *n* = 5–6 mice per group, *p < 0.05, Student´s unpaired *t* test

Given the close link between mPTP opening and calcium overload, we assessed mitochondrial calcium handling by measuring CRC and the protein levels of the main mitochondrial calcium channel, MCU, and its regulator, MICU1. Isolated mitochondria were gradually loaded with small amounts of calcium until mPTP opened and mitochondria ruptured suddenly. To evaluate mPTP sensitivity, half of the mitochondrial samples were treated with CsA, which delays mPTP opening and thereby increases CRC. We observed increased CRC in all groups after administration of CsA. No other significant differences were detected among the experimental groups (Fig. 7a and b). Regarding protein level, a significant increase of MCU was present in the chronically hypoxic WT group compared to normoxic control and chronically hypoxic *Hif1a*^*+/−*^ groups (Fig. 7c), while its regulator MICU1 remained unchanged (Fig. 7d). These results suggest that HIF-1α alters the abundance of MCU; however, this change does not appear to play a major role in mitochondrial calcium retention under the tested conditions.

### HIF-1α inhibition increased cell death after oxidative stress exposure

**Fig. 8:**
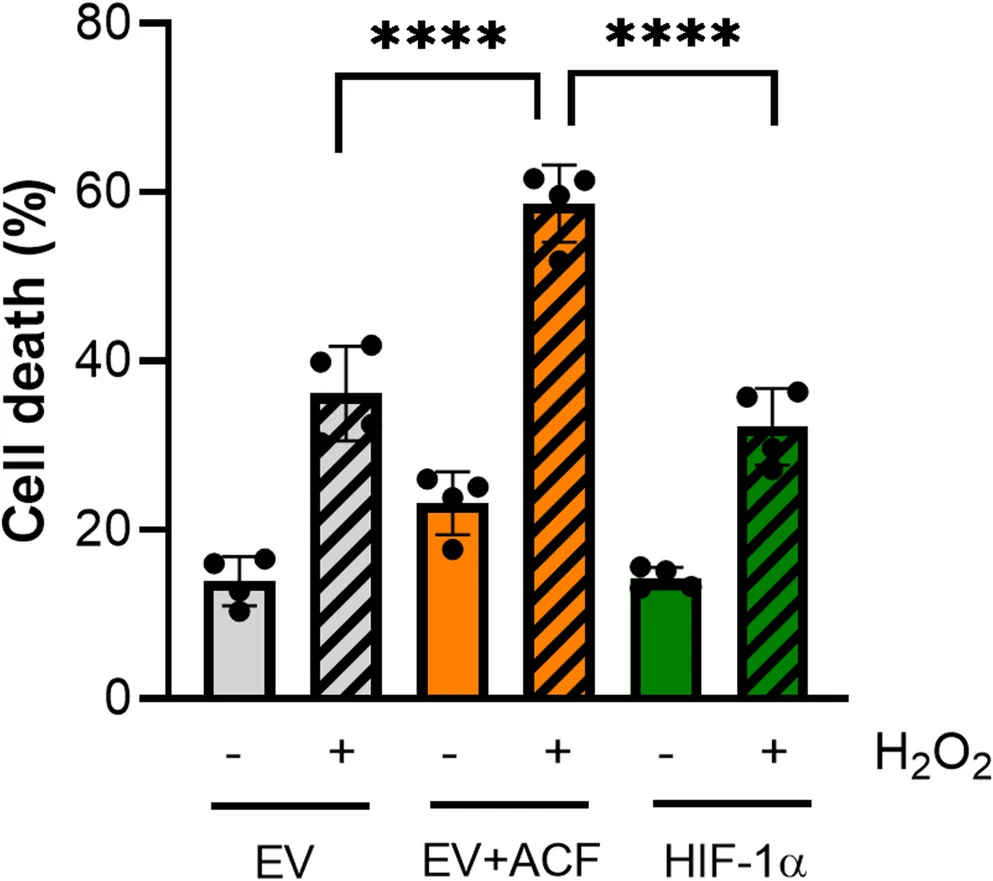
HIF-1α inhibition increased cell death after oxidative stress. Measurement of cell death after exposure to 200 µM H_2_O_2_ for 4 h in the AC16 cells transfected with empty vector (EV) or HIF-1α overexpressing plasmid (HIF-1α). Cells containing EV were treated or untreated by acriflavine (ACF). Values are means ± SD, *n* = 4 separately transfected cell lines per group, ****p < 0.0001, two-way ANOVA with Bonferroni’s multiple comparisons test

To test whether HIF-1α is a key regulator of cardiomyocyte response to oxidative stress, we performed a proof-of-concept study in the human cardiomyocyte AC16 cell line with either HIF-1α suppression or overexpression (Fig. 8). The cells were exposed to H_2_O_2_ to simulate oxidative stress during I/R insult. Additionally, the cells containing EV were untreated or treated with ACF, the most potent HIF-1 inhibitor [28]. Cell death was measured as LDH release in the collected samples. Inhibition of HIF-1α resulted in a significant increase in cell death upon exposure to H_2_O_2_ compared with control cells and HIF-1α-overexpressing cells. Therefore, we can conclude that HIF-1α improves cardiomyocyte survival under conditions of oxidative stress.

## Discussion

In the present study, we investigated the contribution of HIF-1α signaling to the adaptive response of chronically hypoxic mouse hearts to I/R insult, with special regard to oxidative stress regulation, mitochondrial function, and mPTP. Our data demonstrate that HIF-1α mediates CH-induced cardioprotection against I/R injury by attenuating oxidative stress. Moreover, HIF-1α deficiency was associated with altered levels of mPTP-related proteins and a reduced translocation of HXK2 to mitochondria, suggesting that HIF-1α preserves mitochondrial integrity and modulates mPTP susceptibility under hypoxic conditions.

### HIF-1α and myocardial protection against I/R injury

Cardiovascular diseases are frequently accompanied by mitochondrial dysfunction, making mitochondria an important target for cardioprotective interventions. We have previously demonstrated that adaptation to CH also affects mitochondrial function [17, 29]. Both ischemic preconditioning and postconditioning [30, 31], as well as adaptation to CH [16], have been linked to mPTP inhibition. In the present study, we confirmed that CsA reduced infarct size in normoxic hearts. However, it did not provide any additional protective effect beyond that induced by CH in WT mice. In contrast, neither CH nor CsA affected infarct size in *Hif1a*^*+/−*^ hearts. These results suggest that HIF-1α mediates CH-induced cardioprotection by regulating mPTP opening. The protective role of HIF-1α against acute I/R injury is in line with previous studies. Semenza’s group [32] demonstrated that heterozygous *Hif1a* knockout mice lost the reduction in myocardial infarct size afforded by intermittent hypoxia pretreatment. They also found that intraperitoneal administration of erythropoietin, a HIF-1 target gene product, improved post-ischemic cardiac function in HIF-1α-deficient mice. Furthermore, normoxic HIF-1α stabilization reduces infarct size in rats via the prolyl hydroxylase inhibitor GSK360A [33].

### HIF-1α and proteome

HIF-1α was reported to induce the expression of target genes such as pyruvate dehydrogenase kinase 1, BNIP3, and a switch from the cytochrome *c* oxidase subunit COX4-1 to COX4-2 to protect the heart by reducing ROS production [34]. We performed quantitative proteomic profiling with targeted protein analyses to assess the impact of CH and *Hif1a* deficiency on the cardiac proteome. The affected pathways were directly related to mitochondrial function and energy metabolism. In WT hearts, CH induced upregulation of proteins linked to antioxidant defense, HIF-1α signaling, and cellular protection, alongside downregulation of proteins related to fatty acid oxidation, and oxidative phosphorylation, consistent with metabolic remodeling. In contrast, chronically hypoxic *Hif1a*^*+/−*^ exhibited suppression of proteins associated with mitochondrial function, energy metabolism, and HIF-1α signaling. We also identified downregulation of β-hydroxybutyrate dehydrogenase (BDH) in chronically hypoxic *Hif1a*^*+/−*^ mice compared with the corresponding WT group. BDH is a crucial enzyme in ketone metabolism, catalyzing the conversion of 3-hydroxybutyrate (3-OHB) to acetoacetate. Ma et al. reported that 3-OHB suppresses HIF-1α expression under hypoxic conditions in cardiomyocytes and thereby regulates glycolysis-related proteins [35]. Reduced BDH expression in *Hif1a*^*+/−*^ hearts may therefore indicate a reduced metabolic flexibility, limiting the capacity to switch between energy substrates under hypoxic conditions.

### HIF-1α and ROS

Activation of HIF-1α under hypoxic or ischemic conditions promotes metabolic reprogramming from oxidative phosphorylation toward glycolysis, which reduces mitochondrial oxygen consumption, limits electron transport chain overload, and decreases ROS generation - a major trigger of mPTP opening [36]. While excessive ROS levels are a major source of cellular damage, they might act as important signaling molecules [37]. In physiological systems, ROS are produced in balance with the antioxidant system.

Our present study showed enhanced antioxidant defense, as evidenced by reduced oxidative stress markers and moderately increased content of some antioxidant enzymes in chronically hypoxic WT mice. Previous studies in various rat models have yielded diverse results [38, 39]. The importance of antioxidant defense is highlighted by Kasparova et al., who studied the effect of three different regimens of adaptation to CH in rats [40]. In cardioprotective regimens, the majority of mRNAs for antioxidant genes involved in mitochondrial antioxidant defense (e.g., SODM, glutathione reductase, and PRDX2) were upregulated. The nonprotective regimen of continuous normobaric hypoxia (FIO_2_ 0.1, 23 h/day, 3 weeks) increased only PRX5, which was not sufficient to induce the cardioprotective phenotype. In contrast, unchanged protein levels of SODM and CAT in LV myocardium were found using a protective protocol of intermittent hypobaric hypoxia in rats [37]. Together, these findings suggest that upregulation of antioxidant gene expression does not necessarily result in increased protein levels during CH-induced cardioprotection.

ROS have been reported to affect HIF-1 activity. Prolyl hydroxylases, negative regulators of HIF-1α expression under normoxia, were shown to be inactivated by ROS [41]. Importantly, HIF-1α stabilization under normoxia has been shown to decrease mitochondrial ROS in isolated cardiomyocytes and to inhibit mPTP opening in intact rat hearts [33]. HIF-1α also modulates ROS by regulating mitochondrial respiratory function, activating signaling pathways such as PI3K/AKT and JAK2/STAT3 during I/R injury [42]. Semenza [43] reviewed the complex role of HIF-1 in ROS suppression under hypoxic conditions, leading to stabilization of mitochondrial function.

A recent study showed protective HIF-1α translocation to mitochondria, upon exposure to oxidative stress in various human cell lines [44]. This finding is consistent with our results, in which HIF-1α overexpression reduced cell death in AC16 cardiomyocytes exposed to H_2_O_2_. This protective effect also occurred in EV cells, likely due to basal HIF-1α expression. Proliferating normoxic cells can express basal HIF-1α due to growth-factor-dependent signaling and metabolic demands, even in the absence of hypoxia, reflecting developmental and metabolic programming rather than hypoxic stabilization [45]. This supports the role of HIF-1α in regulating mitochondrial function, redox balance, and cellular maturation independently of oxygen deprivation. Here, we showed that HIF-1α activated antioxidant enzymes to limit excessive ROS production and thus, improved cardiomyocyte survival.

### HIF-1α and mPTP

Although the precise molecular identity of mPTP remains unknown, both ANT and the mitochondrial F_1_/F_O_-ATP synthase have been implicated [46]. In the case of F_1_/F_O_-ATP synthase, two hypotheses about the mechanisms of pore formation were proposed. In the first, channel formation would require dimers [47, 48]. In the second hypothesis, the channel would be formed within the c-ring of monomers with conformational effects transmitted by peripheral stalk subunits such as OSCP [49]. We demonstrated that CH tended to increase the content of ATP5I, ATP5L, and ATPMD - proteins involved in F_1_/F_O_-ATP synthase dimerization. Moreover, our proteomic data identified downregulation of the ATPO protein, representing the OSCP subunit of F_1_/F_O_-ATP synthase, in chronically hypoxic mice. This subunit has been identified as a binding site for CYPD [50]. Although the protein level of the main mPTP regulator, CYPD, remained unchanged, CH-induced protection modulated mPTP opening by downregulating its binding partner ATPO and increasing proteins involved in F_1_/F_O_-ATP synthase dimerization.

Further, we investigated whether HIF-1α alters the abundance of other suggested mPTP-related proteins. While CYPD, VDAC, and BAX did not show changes in protein levels, we observed HIF-1α-dependent translocation of HXK2 to mitochondria. HXK2, a downstream target of HIF-1α, is one of the main regulators of mPTP opening. Several studies have demonstrated that mitochondrial-bound HXK2 is a critical determinant of infarct size, and that increasing mitochondrial HXK2 binding during ischemia and early reperfusion contributes to cardioprotection [51]. Increased HXK activity was reported in the mitochondrial fraction after adaptation to continuous normobaric hypoxia in spontaneously hypertensive rats, and this activity negatively correlated with infarct size [52].

### HIF-1α and mitochondrial calcium

Mitochondrial calcium handling is critical for cardiomyocyte survival, particularly under stress conditions where calcium overload can trigger mPTP opening and subsequent cell death. As expected, inhibition of mPTP by CsA significantly increased CRC across all experimental groups. However, no differences in CRC were observed between normoxic and chronically hypoxic conditions. Although somewhat unexpected, this finding is consistent with the concept that mPTP regulation is multifactorial and not solely determined by mitochondrial calcium uptake capacity.

In principle, sustained mitochondrial calcium elevation could contribute to oxidative stress [53]. ROS generated during the adaptation period are important components of cardioprotective signaling induced by CH [37], which have also been reported to stabilize HIF-1α protein [41]. Previous studies suggest a bidirectional relationship between mitochondrial calcium handling and HIF-1α signaling. Tosatto et al. [54] demonstrated that silencing MCU in breast cancer cells reduces HIF-1α transcription, supporting the idea that mitochondrial calcium uptake maintains HIF-1α activity. We also observed increased MCU expression in chronically hypoxic hearts. Furthermore, recent work by Zaglia et al. showed that MCU overexpression prolongs the adaptive phase of cardiac hypertrophy following transverse aortic constriction, likely via enhanced mitochondrial calcium uptake and ROS-dependent activation of Akt signaling [55]. Recent findings by Choya-Foces et al. highlight that mitochondrial calcium uptake and mPTP sensitivity are tightly regulated by redox-dependent mechanisms, rather than by MCU abundance alone [56]. Their study demonstrates that oxidative modifications can modulate mitochondrial calcium fluxes and downstream signaling without necessarily altering CRC. This supports our observation that increased MCU protein levels in chronically hypoxic hearts do not translate into changes in CRC.

### Limitations of the study

The study has several limitations. Obviously, it is necessary to note that differences across the experimental studies may be due to the use of various animal species, strains, and hypoxic protocols as sensitivity to hypoxia shows marked interspecies variability. Mice are generally more tolerant to hypoxia than humans and rats [57]. In addition, the use of heterozygous *Hif1a* knockout mice - necessitated by the non-viability of homozygous deletion - does not fully reflect a complete loss of function, which could also influence the observed phenotype. Among the possible hypoxic protocols, we selected a well-established and reproducible protocol of severe intermittent hypoxia that induces long-lasting cardioprotection in rats [13], which we have previously shown to be effective in mice as well [17]. However, we cannot exclude that modification of this protocol (e.g., duration or severity) might elicit a stronger hypoxic response.

Finally, multiple-testing correction was not applied to the Volcano plot analysis. Importantly, the observed protein changes were principally used to identify biological pathways and functional processes potentially affected by the experimental model. These findings guided subsequent analyses, in which selected targets and pathways were further validated using independent experimental approaches.

Although our data support a role for HIF-1α in modulating mPTP-related pathways, the involvement of mPTP in the observed cardioprotection is based on indirect evidence (protein content and HXK2 translocation), and direct functional confirmation will require further investigation.

## Conclusions

Together, our findings identify HIF-1α as a key mediator of cardioprotection induced by CH against I/R injury, acting through enhanced mitochondrial translocation of HXK2 and attenuation of oxidative stress, suggesting maintained mitochondrial integrity and reduced susceptibility to mPTP opening. These findings suggest that targeting HIF-1α signaling could be a promising therapeutic strategy to enhance mitochondrial resilience and reduce myocardial injury during I/R.

## Data Availability

The data generated during the current study are openly available in the Zenodo repository, https://zenodo.org/records/16963740.

## Abbreviations

ACF: Acriflavine
ANT: Adenine nucleotide translocator
ATPB / ATPO / ATP5I / ATP5L / ATP5H / ATPMD / ATP5J / AT5F1 / ATPA / ATPG: Subunits of F₁/F₀-ATP synthase
BAX: Bcl-2-associated X protein
BDH: β-hydroxybutyrate dehydrogenase
BNIP3: Bcl-2 19-kDa interacting protein 3
CAT: Catalase
CH: Chronic hypoxia
COX: Cytochrome *c* oxidase
CRC: Calcium retention capacity
CsA: Cyclosporine A
CYPD: Cyclophilin D
DMEM: Dulbecco’s Modified Eagle Medium
DMSO: Dimethyl sulfoxide
EDTA: Ethylenediaminetetraacetic acid
ELISA: Enzyme-linked immunosorbent assay
ENOB: β-enolase
ESI: Electrospray ionization
EV: Empty vector
FBS: Fetal bovine serum
FC: Fold change
FDR: False discovery rate
GO: Gene Ontology
GPX4: Glutathione peroxidase 4
GSTM1 / GSTM2: Glutathione S-transferase Mu 1 / 2
H₂O₂: Hydrogen peroxide
HIF-1α: Hypoxia-inducible factor-1 alpha
HXK2: Hexokinase-2
I/R: Ischemia/reperfusion
IMM: Inner mitochondrial membrane
JAK2/STAT3: Janus kinase/Signal transducer and activator of transcription
LC: MS-Liquid chromatography-mass spectrometry
LDH: Lactate dehydrogenase
LV: Left ventricle
MDA: Malondialdehyde
MICOS: Mitochondrial contact sites
MICU1: Mitochondrial calcium uptake 1
MCU: Mitochondrial calcium uniporter
MPCP: Mitochondrial phosphate carrier
mPTP: Mitochondrial permeability transition pore
MS: Mass spectrometry
3-NT: 3-nitrotyrosine
3-OHB: 3-hydroxybutyrate
PARK7: Parkinson disease protein 7 homolog
PBS: Phosphate-buffered saline
PI3K/AKT: Phosphatidylinositol 3 kinase/Protein kinase B
PRDX: Peroxiredoxin
PPI: Protein-protein interaction
ROS: Reactive oxygen species
RV: Right ventricle
SODC: Cytosolic superoxide dismutase
SODM: Mitochondrial superoxide dismutase
STE: Sodium Chloride-Tris-EDTA
STRING: Search Tool for the Retrieval of Interacting Genes/Proteins
TCA: Tricarboxylic acid cycle
TES: N-[Tris(hydroxymethyl)methyl]-2-aminoethanesulfonic acid
TFAM: Mitochondrial transcription factor A
TSPO: Translocator protein
TTC: 2,3,5-triphenyltetrazolium chloride
VDAC: Voltage-dependent anion channel
WT: Wild type

## References

[1] Hausenloy DJ, Duchen MR, Yellon DM (2003) Inhibiting mitochondrial permeability transition pore opening at reperfusion protects against ischaemia-reperfusion injury. Cardiovasc Res 60:617–625. 10.1016/j.cardiores.2003.09.025 14659807

[2] Paillard M, Abdellatif M, Andreadou I, Bär C, Bertrand L, Brundel B, Chiva-Blanch G, Davidson SM, Dawson D, Di Lisa F, Evans P, Giricz Z, Hausenloy DJ, Kleinbongard P, Lezoualc’h F, Liehn E, Maack C, Maguy A, Murphy E, Perrino C, Pesce M, Rainer PP, Streckfuss-Bömeke K, Thielmann M, Tian R, Tocchetti CG, Van Der Velden J, Van Linthout S, Zacchigna S, Krieg T (2025) Mitochondrial targets in ischaemic heart disease and heart failure, and their potential for a more efficient clinical translation. A scientific statement of the ESC Working Group on Cellular Biology of the Heart and the ESC Working Group on Myocardial Function. Eur J Heart Fail. 10.1002/ejhf.3674 40320260 PMC12502468

[3] Griffiths EJ, Halestrap AP (1995) Mitochondrial non-specific pores remain closed during cardiac ischaemia, but open upon reperfusion. Biochem J 307(Pt 1):93–98. 10.1042/bj3070093 7717999 PMC1136749

[4] Baines CP, Kaiser RA, Purcell NH, Blair NS, Osinska H, Hambleton MA, Brunskill EW, Sayen MR, Gottlieb RA, Dorn GW, Robbins J, Molkentin JD (2005) Loss of cyclophilin D reveals a critical role for mitochondrial permeability transition in cell death. Nature 434:658–662. 10.1038/nature03434 15800627

[5] Szabó I, De Pinto V, Zoratti M (1993) The mitochondrial permeability transition pore may comprise VDAC molecules. II. The electrophysiological properties of VDAC are compatible with those of the mitochondrial megachannel. FEBS Lett 330:206–210. 10.1016/0014-5793(93)80274-x 7689984

[6] Kwong JQ, Davis J, Baines CP, Sargent MA, Karch J, Wang X, Huang T, Molkentin JD (2014) Genetic deletion of the mitochondrial phosphate carrier desensitizes the mitochondrial permeability transition pore and causes cardiomyopathy. Cell Death Differ 21:1209–1217. 10.1038/cdd.2014.36 24658400 PMC4085527

[7] Patel P, Mendoza A, Robichaux DJ, Wang MC, Wehrens XHT, Karch J (2021) Inhibition of the Anti-Apoptotic Bcl-2 Family by BH3 Mimetics Sensitize the Mitochondrial Permeability Transition Pore Through Bax and Bak. Front Cell Dev Biol 9:765973. 10.3389/fcell.2021.765973 34926454 PMC8672142

[8] Pastorino JG, Simbula G, Gilfor E, Hoek JB, Farber JL (1994) Protoporphyrin IX, an endogenous ligand of the peripheral benzodiazepine receptor, potentiates induction of the mitochondrial permeability transition and the killing of cultured hepatocytes by rotenone. J Biol Chem 269:31041–31046 7983042

[9] Urbani A, Giorgio V, Carrer A, Franchin C, Arrigoni G, Jiko C, Abe K, Maeda S, Shinzawa-Itoh K, Bogers JFM, McMillan DGG, Gerle C, Szabò I, Bernardi P (2019) Purified F-ATP synthase forms a Ca(2+)-dependent high-conductance channel matching the mitochondrial permeability transition pore. Nat Commun 10:4341. 10.1038/s41467-019-12331-1 31554800 PMC6761146

[10] Karch J, Bround MJ, Khalil H, Sargent MA, Latchman N, Terada N, Peixoto PM, Molkentin JD (2019) Inhibition of mitochondrial permeability transition by deletion of the ANT family and CypD. Sci Adv 5:eaaw4597. 10.1126/sciadv.aaw4597 31489369 PMC6713508

[11] Di Lisa F, Bernardi P (2009) A CaPful of mechanisms regulating the mitochondrial permeability transition. J Mol Cell Cardiol 46:775–780. 10.1016/j.yjmcc.2009.03.006 19303419

[12] Antonucci S, Di Sante M, Sileikyte J, Deveraux J, Bauer T, Bround MJ, Menabò R, Paillard M, Alanova P, Carraro M, Ovize M, Molkentin JD, Cohen M, Forte MA, Bernardi P, Di Lisa F, Murphy E (2020) A novel class of cardioprotective small-molecule PTP inhibitors. Pharmacol Res 151:104548. 10.1016/j.phrs.2019.104548 31759087 PMC8500536

[13] Neckár J, Ostádal B, Kolár F (2004) Myocardial infarct size-limiting effect of chronic hypoxia persists for five weeks of normoxic recovery. Physiol Res 53:621–628 15588130

[14] Wang GL, Jiang BH, Rue EA, Semenza GL (1995) Hypoxia-inducible factor 1 is a basic-helix-loop-helix-PAS heterodimer regulated by cellular O2 tension. Proc Natl Acad Sci U S A 92:5510–5514. 10.1073/pnas.92.12.5510 7539918 PMC41725

[15] Knutson AK, Williams AL, Boisvert WA, Shohet RV (2021) HIF in the heart: development, metabolism, ischemia, and atherosclerosis. J Clin Invest 131. 10.1172/jci137557 PMC8409592 34623330

[16] Alanova P, Alan L, Neckar J, Ostadal B, Kolar F (2024) Cardioprotective Effect of Chronic Hypoxia Involves Inhibition of Mitochondrial Permeability Transition Pore Opening. Physiol Res 73:881–884. 10.33549/physiolres.935427 39560196 PMC11629960

[17] Alanova P, Alan L, Opletalova B, Bohuslavova R, Abaffy P, Matejkova K, Holzerova K, Benak D, Kaludercic N, Menabo R, Di Lisa F, Ostadal B, Kolar F, Pavlinkova G (2024) HIF-1α limits myocardial infarction by promoting mitophagy in mouse hearts adapted to chronic hypoxia. Acta Physiol (Oxf) 240:e14202. 10.1111/apha.14202 39016532

[18] Ong SB, Subrayan S, Lim SY, Yellon DM, Davidson SM, Hausenloy DJ (2010) Inhibiting mitochondrial fission protects the heart against ischemia/reperfusion injury. Circulation 121:2012–2022. 10.1161/circulationaha.109.906610 20421521

[19] Bosch-Marce M, Okuyama H, Wesley JB, Sarkar K, Kimura H, Liu YV, Zhang H, Strazza M, Rey S, Savino L, Zhou YF, McDonald KR, Na Y, Vandiver S, Rabi A, Shaked Y, Kerbel R, Lavallee T, Semenza GL (2007) Effects of aging and hypoxia-inducible factor-1 activity on angiogenic cell mobilization and recovery of perfusion after limb ischemia. Circ Res 101:1310–1318. 10.1161/circresaha.107.153346 17932327

[20] Bohuslavová R, Kolář F, Kuthanová L, Neckář J, Tichopád A, Pavlinkova G (2010) Gene expression profiling of sex differences in HIF1-dependent adaptive cardiac responses to chronic hypoxia. J Appl Physiol (1985) 109:1195–1202. 10.1152/japplphysiol.00366.2010 20634361

[21] Andelova N, Waczulikova I, Talian I, Sykora M, Ferko M (2020) mPTP Proteins Regulated by Streptozotocin-Induced Diabetes Mellitus Are Effectively Involved in the Processes of Maintaining Myocardial Metabolic Adaptation. Int J Mol Sci 21. 10.3390/ijms21072622 PMC7177250 32283821

[22] Pecinová A, Drahota Z, Nůsková H, Pecina P, Houštěk J (2011) Evaluation of basic mitochondrial functions using rat tissue homogenates. Mitochondrion 11:722–728. 10.1016/j.mito.2011.05.006 21664301

[23] Alan L, Opletalova B, Hayat H, Markovic A, Hlavackova M, Vrbacky M, Mracek T, Alanova P (2025) Mitochondrial metabolism and hypoxic signaling in differentiated human cardiomyocyte AC16 cell line. Am J Physiol Cell Physiol 328:C1571–c1585. 10.1152/ajpcell.00083.2025 40243908

[24] Szklarczyk D, Gable AL, Lyon D, Junge A, Wyder S, Huerta-Cepas J, Simonovic M, Doncheva NT, Morris JH, Bork P, Jensen LJ, Mering CV (2019) STRING v11: protein-protein association networks with increased coverage, supporting functional discovery in genome-wide experimental datasets. Nucleic Acids Res 47:D607–d613. 10.1093/nar/gky1131 30476243 PMC6323986

[25] Azoulay-Zohar H, Israelson A, Abu-Hamad S, Shoshan-Barmatz V (2004) In self-defence: hexokinase promotes voltage-dependent anion channel closure and prevents mitochondria-mediated apoptotic cell death. Biochem J 377:347–355. 10.1042/bj20031465 14561215 PMC1223882

[26] Waskova-Arnostova P, Elsnicova B, Kasparova D, Hornikova D, Kolar F, Novotny J, Zurmanova J (2015) Cardioprotective adaptation of rats to intermittent hypobaric hypoxia is accompanied by the increased association of hexokinase with mitochondria. J Appl Physiol (1985) 119:1487–1493. 10.1152/japplphysiol.01035.2014 26494452

[27] Nederlof R, Eerbeek O, Hollmann MW, Southworth R, Zuurbier CJ (2014) Targeting hexokinase II to mitochondria to modulate energy metabolism and reduce ischaemia-reperfusion injury in heart. Br J Pharmacol 171:2067–2079. 10.1111/bph.12363 24032601 PMC3976622

[28] Piorecka K, Kurjata J, Stanczyk WA (2022) Acriflavine, an Acridine Derivative for Biomedical Application: Current State of the Art. J Med Chem 65:11415–11432. 10.1021/acs.jmedchem.2c00573 36018000 PMC9469206

[29] Borchert GH, Yang C, Kolár F (2011) Mitochondrial BKCa channels contribute to protection of cardiomyocytes isolated from chronically hypoxic rats. Am J Physiol Heart Circ Physiol 300:H507–H513. 10.1152/ajpheart.00594.2010 21112945 PMC3044046

[30] Hausenloy DJ, Lim SY, Ong SG, Davidson SM, Yellon DM (2010) Mitochondrial cyclophilin-D as a critical mediator of ischaemic preconditioning. Cardiovasc Res 88:67–74. 10.1093/cvr/cvq113 20400621 PMC2936122

[31] Lim SY, Davidson SM, Hausenloy DJ, Yellon DM (2007) Preconditioning and postconditioning: the essential role of the mitochondrial permeability transition pore. Cardiovasc Res 75:530–535. 10.1016/j.cardiores.2007.04.022 17512507 PMC2080572

[32] Cai Z, Manalo DJ, Wei G, Rodriguez ER, Fox-Talbot K, Lu H, Zweier JL, Semenza GL (2003) Hearts from rodents exposed to intermittent hypoxia or erythropoietin are protected against ischemia-reperfusion injury. Circulation 108:79–85. 10.1161/01.Cir.0000078635.89229.8a 12796124

[33] Ong SG, Lee WH, Theodorou L, Kodo K, Lim SY, Shukla DH, Briston T, Kiriakidis S, Ashcroft M, Davidson SM, Maxwell PH, Yellon DM, Hausenloy DJ (2014) HIF-1 reduces ischaemia-reperfusion injury in the heart by targeting the mitochondrial permeability transition pore. Cardiovasc Res 104:24–36. 10.1093/cvr/cvu172 25063991

[34] Semenza GL (2014) Hypoxia-inducible factor 1 and cardiovascular disease. Annu Rev Physiol 76:39–56. 10.1146/annurev-physiol-021113-170322 23988176 PMC4696033

[35] Ma X, Dong Z, Liu J, Ma L, Sun X, Gao R, Pan L, Zhang J, An AD, Hu J, Sun K A and, Ge J (2022) β-Hydroxybutyrate Exacerbates Hypoxic Injury by Inhibiting HIF-1α-Dependent Glycolysis in Cardiomyocytes-Adding Fuel to the Fire? Cardiovasc Drugs Ther 36:383–397. 10.1007/s10557-021-07267-y 34652582 PMC9090701

[36] Semenza GL (2001) Hypoxia-inducible factor 1: control of oxygen homeostasis in health and disease. Pediatr Res 49:614–617. 10.1203/00006450-200105000-00002 11328942

[37] Kolár F, Jezková J, Balková P, Breh J, Neckár J, Novák F, Nováková O, Tomásová H, Srbová M, Ost’ádal B, Wilhelm J, Herget J (2007) Role of oxidative stress in PKC-delta upregulation and cardioprotection induced by chronic intermittent hypoxia. Am J Physiol Heart Circ Physiol 292:H224–H230. 10.1152/ajpheart.00689.2006 16936002

[38] Neckár J, Borchert GH, Hlousková P, Mícová P, Nováková O, Novák F, Hroch M, Papousek F, Ost’ádal B, Kolár F (2013) Brief daily episode of normoxia inhibits cardioprotection conferred by chronic continuous hypoxia. Role of oxidative stress and BKCa channels. Curr Pharm Des 19:6880–6889. 10.2174/138161281939131127115154 23590154

[39] Chytilová A, Borchert GH, Mandíková-Alánová P, Hlaváčková M, Kopkan L, Khan MA, Imig JD, Kolář F, Neckář J (2015) Tumour necrosis factor-α contributes to improved cardiac ischaemic tolerance in rats adapted to chronic continuous hypoxia. Acta Physiol (Oxf) 214:97–108. 10.1111/apha.12489 25760892

[40] Kasparova D, Neckar J, Dabrowska L, Novotny J, Mraz J, Kolar F, Zurmanova J (2015) Cardioprotective and nonprotective regimens of chronic hypoxia diversely affect the myocardial antioxidant systems. Physiol Genomics 47:612–620. 10.1152/physiolgenomics.00058.2015 26465708

[41] Li YN, Xi MM, Guo Y, Hai CX, Yang WL, Qin XJ (2014) NADPH oxidase-mitochondria axis-derived ROS mediate arsenite-induced HIF-1α stabilization by inhibiting prolyl hydroxylases activity. Toxicol Lett 224:165–174. 10.1016/j.toxlet.2013.10.029 24188932

[42] Fuhrmann DC, Brüne B (2017) Mitochondrial composition and function under the control of hypoxia. Redox Biol 12:208–215. 10.1016/j.redox.2017.02.012 28259101 PMC5333533

[43] Semenza GL (2011) Hypoxia-inducible factor 1: regulator of mitochondrial metabolism and mediator of ischemic preconditioning. Biochim Biophys Acta 1813:1263–1268. 10.1016/j.bbamcr.2010.08.006 20732359 PMC3010308

[44] Li HS, Zhou YN, Li L, Li SF, Long D, Chen XL, Zhang JB, Feng L, Li YP (2019) HIF-1α protects against oxidative stress by directly targeting mitochondria. Redox Biol 25:101109. 10.1016/j.redox.2019.101109 30686776 PMC6859547

[45] Lum JJ, Bui T, Gruber M, Gordan JD, DeBerardinis RJ, Covello KL, Simon MC, Thompson CB (2007) The transcription factor HIF-1alpha plays a critical role in the growth factor-dependent regulation of both aerobic and anaerobic glycolysis. Genes Dev 21:1037–1049. 10.1101/gad.1529107 17437992 PMC1855230

[46] Bernardi P, Gerle C, Halestrap AP, Jonas EA, Karch J, Mnatsakanyan N, Pavlov E, Sheu SS, Soukas AA (2023) Identity, structure, and function of the mitochondrial permeability transition pore: controversies, consensus, recent advances, and future directions. Cell Death Differ 30:1869–1885. 10.1038/s41418-023-01187-0 37460667 PMC10406888

[47] Wagner K, Rehling P, Sanjuán Szklarz LK, Taylor RD, Pfanner N, van der Laan M (2009) Mitochondrial F1Fo-ATP synthase: the small subunits e and g associate with monomeric complexes to trigger dimerization. J Mol Biol 392:855–861. 10.1016/j.jmb.2009.07.059 19635484

[48] Mnatsakanyan N, Llaguno MC, Yang Y, Yan Y, Weber J, Sigworth FJ, Jonas EA (2019) A mitochondrial megachannel resides in monomeric F(1)F(O) ATP synthase. Nat Commun 10:5823. 10.1038/s41467-019-13766-2 31862883 PMC6925261

[49] Carrer A, Tommasin L, Šileikytė J, Ciscato F, Filadi R, Urbani A, Forte M, Rasola A, Szabò I, Carraro M, Bernardi P (2021) Defining the molecular mechanisms of the mitochondrial permeability transition through genetic manipulation of F-ATP synthase. Nat Commun 12:4835. 10.1038/s41467-021-25161-x 34376679 PMC8355262

[50] Gauba E, Chen H, Guo L, Du H (2019) Cyclophilin D deficiency attenuates mitochondrial F1Fo ATP synthase dysfunction via OSCP in Alzheimer’s disease. Neurobiol Dis 121:138–147. 10.1016/j.nbd.2018.09.020 30266287 PMC6250052

[51] Smeele KM, Southworth R, Wu R, Xie C, Nederlof R, Warley A, Nelson JK, van Horssen P, van den Wijngaard JP, Heikkinen S, Laakso M, Koeman A, Siebes M, Eerbeek O, Akar FG, Ardehali H, Hollmann MW, Zuurbier CJ (2011) Disruption of hexokinase II-mitochondrial binding blocks ischemic preconditioning and causes rapid cardiac necrosis. Circ Res 108:1165–1169. 10.1161/circresaha.111.244962 21527739

[52] Nedvedova I, Kolar D, Elsnicova B, Hornikova D, Novotny J, Kalous M, Pravenec M, Neckar J, Kolar F, Zurmanova JM (2018) Mitochondrial genome modulates myocardial Akt/Glut/HK salvage pathway in spontaneously hypertensive rats adapted to chronic hypoxia. Physiol Genomics 50:532–541. 10.1152/physiolgenomics.00040.2017 29676955

[53] De Stefani D, Rizzuto R, Pozzan T (2016) Enjoy the Trip: Calcium in Mitochondria Back and Forth. Annu Rev Biochem 85:161–192. 10.1146/annurev-biochem-060614-034216 27145841

[54] Tosatto A, Sommaggio R, Kummerow C, Bentham RB, Blacker TS, Berecz T, Duchen MR, Rosato A, Bogeski I, Szabadkai G, Rizzuto R and Mammucari C (2016) The mitochondrial calcium uniporter regulates breast cancer progression via HIF-1α. EMBO Mol Med 8:569-85. doi: 10.15252/emmm.201606255 10.15252/emmm.201606255 PMC4864890 27138568

[55] Zaglia T, Campo A, Moro N, Di Mauro V, Borile G, Menabò R, Antonucci S, Poli L, Campesan M, Carullo P, Martinazzi S, Luciani GB, Hammer K, Pesce P, Bariani R, Faggian G, Maier L, Ventura L, De Stefani D, Mammucari C, Rizzuto R, Catalucci D, Di Lisa F, Mongillo M (2025) Enhancement of mitochondrial calcium uptake is cardioprotective against maladaptive hypertrophy by retrograde signaling uptuning Akt. Proc Natl Acad Sci U S A 122:e2402639122. 10.1073/pnas.2402639122 40067892 PMC11929399

[56] Choya-Foces C, Navarro E, Ríos CL, López MG, Egea J, Hernansanz-Agustín P, Martínez-Ruiz A (2024) The mitochondrial Na(+)/Ca(2+) exchanger NCLX is implied in the activation of hypoxia-inducible factors. Redox Biol 77:103364. 10.1016/j.redox.2024.103364 39341036 PMC11470253

[57] Burtscher J, Mallet RT, Sah A, Gassmann M, Burtscher M, Iturriaga R (2025) Physiological Differences Underlying Divergent Hypoxia Responses and Altitude Adaptations in Humans, Rats and Mice. Compr Physiol 15:e70077. 10.1002/cph4.70077 41307193 PMC12658720

